# Translucency perception: A review

**DOI:** 10.1167/jov.21.8.4

**Published:** 2021-08-03

**Authors:** Davit Gigilashvili, Jean-Baptiste Thomas, Jon Yngve Hardeberg, Marius Pedersen

**Affiliations:** 1 Norwegian University of Science and Technology, Department of Computer Science, Gjøvik, Norway

**Keywords:** translucency perception, material appearance

## Abstract

Translucency is an optical and a perceptual phenomenon that characterizes subsurface light transport through objects and materials. Translucency as an optical property of a material relates to the radiative transfer inside and through this medium, and translucency as a perceptual phenomenon describes the visual sensation experienced by humans when observing a given material under given conditions. The knowledge about the visual mechanisms of the translucency perception remains limited. Accurate prediction of the appearance of the translucent objects can have a significant commercial impact in the fields such as three-dimensional printing. However, little is known how the optical properties of a material relate to a perception evoked in humans. This article overviews the knowledge status about the visual perception of translucency and highlights the applications of the translucency perception research. Furthermore, this review summarizes current knowledge gaps, fundamental challenges and existing ambiguities with a goal to facilitate translucency perception research in the future.

## Introduction

How different objects and materials appear to human observers is important not only in commerce, where customer choice and satisfaction are often influenced by the visual look of the product, but also in trivial daily tasks performed by humans. For instance, we use the visual appearance information to judge whether materials are fragile or elastic, whether food is spoiled or edible. By their appearance, we can effortlessly identify materials within seconds (Sharan et al., 2009; Wiebel et al., 2013). According to the International Commission on Illumination (the CIE - Commission Internationale de l'Eclairage) total appearance “points out the visual aspects of objects and scenes” (Pointer, 2006). Translucency is among the most essential visual attributes of appearance, along with color, gloss, and texture (Pointer, 2006; Eugène, 2008), remaining the least studied one among those (Anderson, 2011). Although the color information incident on the human retina encodes important information about the objects and materials, overall sensation also depends “on the appearance of that colour due to the relationship between the light transmitted, the light reflected, and the light scattered by the body of the object” (Pointer, 2003). Translucency is seen as a phenomenon “between the extremes of complete transparency and complete opacity” (Eugène, 2008). According to the ASTM (2017) translucency is “the property of a specimen by which it transmits light diffusely without permitting a clear view of objects beyond the specimen and not in contact with it.”

The etymology of the term is related to the Latin words “trans” (through) and “lux” (light) — implying light penetration inside the body of the material (Kaltenbach, 2012). A translucent appearance is usually the result of a visual stimulus incident onto a retina from the objects permitting some degree of the *subsurface light transport*. Translucency is impacted by multiple intrinsic and extrinsic factors. The intrinsic factors are the physical parameters found in the *radiative transfer equation* (Chandrasekhar, 1960), such as the index of refraction, and the absorption and scattering coefficients, as well as the scattering phase function. They define how the light propagates through the media. A photon can get absorbed or scattered, that is, redirected toward a different direction when there is a change in the index of refraction, either at the external surface of the object, or inside its volume (Tavel, 1999). How this passage of light through a material relates to a visual sensation of translucency remains unclear to date. The extrinsic factors include, but are not limited to, the illumination direction (Fleming & Bülthoff, 2005; Xiao et al., 2014), object shape (Fleming & Bülthoff, 2005; Gigilashvili et al., 2018b), and the color of the surface a translucent object is placed on (Gigilashvili et al., 2020a). The human visual system (HVS) is remarkably good at detecting subsurface light transport — we can easily tell the difference between a translucent glass and an opaque metal, translucent wax and opaque stone. We can distinguish translucent human skin from an opaque plastic dummy, translucent milk from opaque chalk. One of the fundamental problems is to understand how the HVS interprets the surface-reflected and subsurface-scattered light from the stimuli incident on the human retina. The exact visual and cognitive mechanisms of this ability are far from being fully understood. Because no model has yet been able to predict perceived translucency of a given material in an accurate and robust manner, translucency perception remains a topic of active research in academia and industry alike.

We would like to highlight that the primary focus of this article is *translucency*, not *transparency* — a better understood concept and visual attribute. While the two concepts are sometimes used interchangeably (e.g., Merriam-Webster Dictionary, n.d.), it is usually accepted that *transparent substances, unlike translucent ones, transmit light without diffusing it* (Gerbino et al., 1990). According to the CIE, “if it is possible to see an object through a material, then that material is said to be transparent. If it is possible to see only a “blurred” image through the material (due to some diffusion effect), then it has a certain degree of transparency and we can speak about translucency” (Eugène, 2008). This implies that a given material might possess some degree of transparency and some degree of translucency at the same time.

The contribution of this article is three-fold:

(1) Summarize the state-of-the-art about the perception of translucency and the review of the recent developments in the field.
(2) Discuss the different applications that could benefit from the translucency perception research and overview of the importance of the topic in and across different disciplines.
(3) Outline the major knowledge gaps and research challenges in order to facilitate future work.

The article is organized as follows. We briefly summarize the motivation for translucency perception research in the next section. In four subsequent sections, we review the state of the art and demonstrate the findings on the example of real and synthetic stimuli. First, we provide a historical discourse on how the knowledge status has developed over time. Second, we overview the role of transparency in translucency perception. Third, we discuss which factors impact perceived translucency. Fourth, the potential cues for translucency perception are analyzed. Afterward, we discuss the current challenges in the translucency perception research and outline the most important questions remaining open, which is followed by a concluding section.

## Background and motivation

Translucency plays a significant role in a multitude of fields and applications. Thus, it is a research interest in different disciplines. In this section, we provide an overview of the applications and the interdisciplinarity of the problem. Afterward, we discuss the gap between the optical and the perceptual properties of a material — motivating the research from the human vision point of view.

### Applications

To highlight the importance of understanding underlying visual mechanisms of translucency perception, we summarize the major applications where the translucency perception research can make impact.

A broad range of customer products look translucent, either customers expecting a translucent look from the products, or the degree of translucency itself can be an indicator of product's quality. This raises the need for studying translucency in the respective industries. For example, the foods, such as beer, meat and dairy products, are translucent. Therefore, translucent appearance plays an important role in the **food industry**, not only impacting customer satisfaction (Hutchings, 1977, 2011), but also contributing to the safety assurance (Chousalkar et al., 2010; Ray & Roberts, 2013). **Decorative paint manufacturing** is another example, because the hiding power of the colorants impacts the appearance and the overall quality of the paints (Krewinghaus, 1969; Midtfjord et al., 2018; Zhao & Berns, 2009).

Translucency has an implication for aesthetic purposes as well. Generation, reproduction, and perception of translucent appearance has long been a topic of interest in **visual arts** and **cultural heritage**. Translucent building materials play an important role in the modern-day **architecture** and are used to generate various visual effects of the exterior as well as interior **design** (Kaltenbach, 2012; Murray, 2013). The translucent look of a marble makes it an appealing material actively used both in architecture and **sculptures** (Barry, 2011), whereas the translucency of glass is widely taken advantage of in the **glass art** (Kaltenbach, 2012). A special case is **painting**, where the tradition of photorealistic depiction of the scenes exists from the medieval times and, even though the scenes do not conform to the laws of physics, the artists still have been capable of generating vividly impressive and realistic depictions of the environment (Cavanagh, 2005), seemingly following the rule-of-thumb, heuristic “recipes” (Di Cicco et al., 2020a). Recently, several studies have addressed perception of **painterly materials** (van Zuijlen et al., 2020) with an emphasis on translucency in the **marine art** (Wijntjes et al., 2020) and **still life paintings** (Di Cicco et al., 2020a, 2020c). Translucency is an important attribute for perception of visual realism and aesthetics of the artworks, especially those depicting sea scenes, fruits, and human skin. Understanding how painters generate the vivid sensation of translucency without conforming to the laws of physics can reveal interesting perceptual mechanisms of the HVS. This demonstrates that in addition to the physically based simulations of the visual stimuli in computer graphics, translucency perception research can also greatly benefit from studying artworks, and vice versa.

Translucent appearance is also actively studied in the **aesthetic medicine** and **cosmetology**. The interdisciplinary works in **material science** and **dentistry** emphasize the importance of proper translucent look of the dental implants and restorative materials (Anfe et al., 2008; Liu et al., 2010; Lopes Filho et al., 2012; Seghi et al., 1989 Wilson & Kent, 1971). On the other hand, face powders and moisturizers are used to enhance an appealing translucent look of the human skin (Emmert, 1996; Giancola & Schlossman, 2015), which can be studied by simulation of cosmetics and human skin rendering (Li et al., 2018) in **computer graphics**.

Although **computer graphics** is often used as a tool for studying translucency perception (e.g., Urban et al., 2019; Xiao et al., 2014), perceiving translucency and accurate reproduction of translucent appearance is itself an important topic for the computer graphics community, especially when photorealism is at stake (Frisvad et al., 2020). One of the most significant, yet challenging, topics is accurate rendering of the human skin, which not only plays an essential role in the movies, video games, and other segments of the entertainment industry, but also extends to the fields of computer vision (face detection and edge detection; Gkioulekas et al., 2015), medicine, and cosmetology (Igarashi et al., 2005; Li et al., 2018). Although considerable progress has been made in this direction, **skin rendering**, which inherently implies the accurate reproduction of translucent appearance, is a topic of active ongoing research (d’Eon & Irving, 2011) and remains especially challenging owing to the multilayer nature of a human skin (Frisvad et al., 2020; Nunes et al., 2019).

One of the most novel fields which can benefit from translucency perception research is three-dimensional (**3D**) **printing**. Three-dimensional printing technologies have reached a level of development where translucency has become an important visual attribute, increasingly attracting an attention in the 3D printing community. The recent advances in multimaterial 3D printing enable generation and reproduction of material translucence by mixing transparent and colored opaque printing materials, which expands the appearance gamut of the 3D printing hardware (Brunton et al., 2018). However, object shape and scale dramatically impact perceived translucency, for example, smaller objects transmit more light than the larger objects made of the identical material. To obtain a desired translucent look, mixing ratios of the printing materials should be adapted to these extrinsic factors, which itself needs a deeper understanding of the translucency perception process (Urban et al., 2019). A seminal contribution to this direction has been made by Urban et al. (2019), who proposed a hardware- and software-independent perceptual translucency metric for the 3D printing applications.

These fields might have established their own standards for measuring particular optical properties of the light permeable materials, such as scattering and extinction coefficients. However, the research on translucency perception is needed to understand how those objective measures can be used to predict what the customers will see. Moreover, the measurements are usually done for a small number of predefined shapes, conditions, and geometries, which might not correspond with the real-life encounters and might generalize poorly. Therefore, it is important to know in what way customers’ perception is affected by the extrinsic factors, such as the shape of the object, illumination direction, or motion. Understanding translucency perception and its contributing factors will make replication and matching of the total appearance easier. This will facilitate many appearance-related tasks, such as archiving and conservation in cultural heritage, as well as the development of the perception-aware rendering techniques in computer graphics.

### Physics and perception – The gap

The primary reason why instrumental measurement of the perceptual translucency remains beyond reach is the fact that the definition of the perceptual attributes is vague (see subsection *Inconsistent definition and conceptual ambiguity*) and their physical correlates are not identified. Even though the techniques of material property acquisition have advanced and the photorealism of the computer-generated imagery is impressive, the link between the measured physical properties of the materials and their visual appearance is far from being fully understood. Photosensitive measurement instruments might not be able to capture the appearance perceived by the HVS and cannot provide a quantitative correlate of visual sensation (SABIC Innovative colorXpress, n.d.). In other words, even if we achieve an accurate measurement, modelling and simulation of the optical properties of a given material, we might be able to create a “digital twin” of a real-world object, but we still will not be able to accurately predict how this material, either the real or the virtual, will look to the HVS, limiting our capability to generate desired visual effects from scratch and to replicate the appearance across different objects, scenes and conditions. This largely motivates the attempts of *soft metrology* and the rigorous research on visual appearance in different disciplines.

The knowledge gap is especially apparent when it comes to finding the correlation between the physical properties of subsurface light transport and the perception of material translucence. Although there is a long tradition of research on colors, providing a reasonably deep understanding of color vision and color appearance, the perception of translucency has rarely been explored up until recently.

Indeed, translucency as an optical property of a material can be measured instrumentally (Pointer, 2003). The physical accuracy of rendering in computer graphics is constrained by the accuracy of the input physical material properties, dubbed as “the input problem” by Rushmeier (1995, 2008). This makes accurate measurement of the optical properties especially important. The most comprehensive and up-to-date survey regarding the acquisition of the optical properties of translucent materials has been done by Frisvad et al. (2020).

However, no technique has been proposed to date for an instrumental measurement of perceptual translucency. In other words, we have not been able “to obtain numbers that are representative of the way objects and materials look” (Hunter & Harold, 1987). Multiple application-specific instruments measure transmission-related visual attributes (BYK Gardner GmbH., n.d.), playing an important role in a broad range of industries, from solar cell manufacturing (Preston et al., 2013) to petroleum and edible product quality assurance (Lovibond Tintometer, n.d.). The two most common attributes studied in relation to translucency are **clarity** — “defined in terms of the ability to perceive the fine detail of images through the material,” and **haze** — “defined as a property of the material whereby objects viewed through it appear to be reduced in contrast” (Pointer, 2003). Haze is usually associated with a wide angle scattering (when the angle between the incident illumination and the transmitted light is more than 2.5°, according to the ASTM D1003-21, 2021) of light that causes blur and loss of contrast of the see-through image, while the clarity usually results from a narrow angle (less than 2.5°) scattering. Analysis of the measurement procedures is beyond the scope of this article, but it is important to highlight that no clear link between translucency as an appearance attribute, on the one hand, and clarity and haze, on the other hand, has been established. Pointer (2003) argues that “the concept of translucency can perhaps be regarded as a descriptor of the combined effects defined above as clarity and haze. This implies that it is a more general term and, perhaps, should be limited to use as a subjective term, keeping clarity and haze as descriptors of objective, or measurable, correlates.” In the subsequent sections, we analyze what we know and do not know about perceiving material translucence.

## Historical discourse

A translucent appearance has long been encapsulated in a more general problem of visual appearance of objects and materials. The early theories of the visual appearance proposed that the HVS might invert optical processes in the scene to deduce the physical material properties and thus, the appearance (DZmura & Iverson, 1993; Pizlo, 2001; Poggio & Koch, 1985). Although this hypothesis is nowadays largely disputed (Chadwick et al., 2019; Fleming & Bülthoff, 2005), it remains debatable to what extent and complexity we can talk about “inverting” and estimating physical properties in the scene (Anderson, 2011). The later works proposed that the HVS might be using the heuristic low-level image cues and statistics (Chadwick & Kentridge, 2015; Fleming & Bülthoff, 2005; Motoyoshi et al., 2007; Motoyoshi, 2010) for assessing material properties, including translucency. According to the recent proposal by Fleming (2014), the HVS might be learning a generative model that predicts the variation of appearance across different natural illumination conditions. The recent developments in the material appearance research include unsupervised machine learning techniques to first predict human perception and then get deeper insight into it (Fleming & Storrs, 2019; Prokott & Fleming, 2019; Storrs & Fleming, 2020; van Assen et al., 2020).

The fact that subsurface light transport plays an important role in visual appearance has been obvious from the very first attempts to measure appearance (Hunter & Harold, 1987). It has been important to understand how the light diminishes when passing through the thin layers of materials that either absorb or scatter light, for instance, when several layers of paint or coatings are applied on a given surface, and how this affects the final color. Multiple models have been proposed in the first half of the twentieth century (using a term *turbid materials*). The Kubelka-Munk theory was one of the most widespread as well as simplest among those (Kubelka, 1931, 1948; Vargas & Niklasson, 1997). Kubelka-Munk coefficients K and S of a given paint film describe the portion of the light that gets absorbed and scattered, respectively, per unit thickness travelled through the paint material (Krewinghaus, 1969). Although it remains used for color matching calculations in the industries handling multilayered thin translucent materials, such as ink and dyed paper manufacturing (Yang & Kruse, 2004), its limitations are noteworthy — the model considers just two fluxes of light travelling upward and downward, and assumes that the light is not scattered laterally (Hašan et al., 2010) (although there have been attempts to extend it to the lateral light transport, Donner & Jensen, 2005). Therefore, this kind of simplified models are not applicable to objects with complex geometry and subsurface light transport. Moreover, they might characterize material properties, but they are not suitable for characterization and prediction of translucency appearance.

Early attempts of studying visual perception of subsurface light transport were limited to perception of transparency, which, in some sense, was used as an umbrella term to describe light transmissive materials. Proposed models consider a target transparent material as a thin filter which modulates the color of the background pattern seen through it and which can be described with a simple algebraic relationship (Beck & Ivry, 1988; Gerbino et al., 1990; Gerbino, 1994; Metelli, 1970, 1974, 1985). However, these models did not account for subsurface scattering. For details on perception, depiction and generation of transparency refer to the reviews in (Fleming & Bülthoff, 2005; Sayim & Cavanagh, 2011; Singh & Anderson, 2002a); regarding the perception of thick, complex-shaped transparent objects see the work by Fleming et al. (2011). Although relatively well-understood, transparency still remains a topic of active research (see Falkenberg & Faul, 2019; Faul & Ekroll, 2012). Object and background separation in transparent materials pose an important challenge in the ever emerging field of computer vision (Anderson, 2011).

Although these works explain the perceptual mechanisms of see-through materials, the background is not always visible through the objects and the cues the HVS relies on for transparency perception are simply absent. This is especially true for the materials with high subsurface scattering, when none of the background can be detected through the object and the luminance gradient on its body is the only indicator that the light penetrates inside the volume. Many materials we interact with on a daily basis, such as wax, marble, textile, meat, cream, or milk, are not see-through and cannot be approximated with the perceptual models of transparency. Therefore, the cues used by the HVS for perceiving translucency of the highly scattering media might be fundamentally different from those of transparency. This gave birth to the translucency perception research as a separate topic from transparency perception. The advances in the translucency perception research can be attributed to the rapid advance in computer graphics (Anderson, 2011; Fleming & Bülthoff, 2005). The difficulty to vary subsurface scattering properties systematically impeded generation of the proper visual stimulus datasets for conducting psychophysical experiments or analyzing image statistics. The progress in modelling subsurface scattering (such as Jensen et al., 2001) made the generation of translucent visual stimuli cheap, fast, and fully controllable.

Koenderink and van Doorn (2001) described that the shading patterns differ dramatically between opaque and translucent media and that the “shape from shading” paradigm, which assumes Lambertian opaque surfaces, is not applicable to translucent objects. They raised an interesting question on how the HVS calculates the shape of the translucent objects and discussed an example of atmospheric objects, such as clouds, where shape judgment is entirely speculative. They used diluted and undiluted milk images to demonstrate how the radiance distribution over the material body depends on the mean free path of the photon (which is calculated as $\frac{1}{\alpha +\sigma }$, where α and σ are absorption and scattering coefficients, respectively). They also pointed out that the appearance of translucent objects varies with the point of observation, because the number of photons emerging from an object body differs among different spacial positions. They also drew a parallel with the painters who are able to render a realistic appearance of translucent objects and argued that humans understand translucency in a qualitative way rather than by the means of calculating underlying physics.

This idea was later augmented by Fleming and Bülthoff (2005) in their seminal work, which paved the way for the last two decades’ translucency perception research. They argued that instead of inverting optics, the HVS relies on the low-level image cues for calculating translucency. They examined and described different factors, such as object scale, color saturation, and the presence of specular reflections, potentially affecting perceived translucency. They identified that some regions, such as edges, contain richer information regarding material translucence. They demonstrated that translucency depends on the illumination geometry and back-lit objects look more translucent. Finally, they analyzed how the candidate image statistics, such as the moments of luminance histogram and intensities of the shadowed regions covary with the illumination geometry.

The intensities of the shadowed regions seem to be one of the most significant visual characteristics differentiating translucent and opaque materials. Motoyoshi (2010) proposed that the HVS might be calculating luminace statistics of the nonspecular regions of the image to understand translucency. The author experimentally demonstrated that blurring and decreasing the contrast in the nonspecular regions of the opaque material generates a translucent look.

Later works attempted to identify the impact of the various intrinsic and extrinsic factors on perceived translucency, such as the role of a scattering phase function (Gkioulekas et al., 2013; Xiao et al., 2014) and illumination direction (Xiao et al., 2014). Further works identified the spatial regions, which are the most informative for understanding translucency (Gkioulekas et al., 2015; Nagai et al., 2013). Similar to Fleming and Bülthoff (2005), Gkioulekas et al. (2015) also observed that edges contain a vital portion of the information about the subsurface light transport and discussed a potential use of the edge profiles as a physical correlate of translucency. Marlow et al. (2017) found that the lack of covariance between the shape and shading information correlates well with the perceptual translucency. They demonstrated that illusory translucency can be evoked on optically opaque objects when the diffuse light field generates the shading that is not covariant with the surface geometry. The study has an interesting implication that translucency perception might be adjoined with the shape perception. The recent study by Chadwick et al. (2019) demonstrated that translucency perception is anatomically independent from color and texture perception.

The rapid development in the 3D printing technologies, which permit accurate generation of the physical objects with complex subsurface light transport properties (Brunton et al., 2018, 2020), on the one hand yielded an opportunity to use the physical objects instead of the computer-generated imagery in psychophysical experiments (Vu et al., 2016), and, on the other hand, increased an industrial demand on the translucency perception research (Gigilashvili et al., 2019c; Urban et al., 2019). Urban et al. (2019) have recently proposed a perceptually uniform measure *Alpha* for 3D printing applications, which can also account for an object scale. Gigilashvili et al. (2018b, 2021b) argued that, when observing displayed images, observers cannot enjoy the fully realistic experience they have on a daily basis when interacting with translucent materials. The authors believe that, although having full control of the scene and the optical parameters, these kind of experiments might not reveal all behavioral patterns and thus, the visual mechanisms for translucency assessment. They used handcrafted physical objects (Thomas et al., 2018) for translucency assessment tasks and analyzed the behavioral patterns qualitatively. They observed that the dynamic cues, such as moving objects in relation with a textured background and head movements, as well as comparison of the given object's appearance between back-lit and front-lit illumination conditions, are used frequently by human observers while judging translucency. They also found that, in addition to the appearance of a given object, the extrinsic cues elsewhere in the scene, such as caustics projected by an object onto a different surface, might also facilitate judgement of translucency (Gigilashvili et al., 2020a). The advantages and disadvantages of using physical and digital stimuli are discussed elsewhere in this article.

## Translucency of see-through media

Transparency, translucency, and opacity relate to the same phenomenon — the subsurface scattering of light (or the lack of thereof). The internal scattering gradually makes a perfectly transparent medium more translucent and eventually opaque (Gerardin et al., 2019; Gigilashvili et al., 2020b). The boundary among them is fuzzy, implying that transparency and translucency are not mutually exclusive. Some degree of transparency and some degree of translucency can coexist in the same stimulus. As noted elsewhere in this article, translucent materials scatter light, whereas perfectly transparent ones do not (Gerbino et al., 1990). However, in some cases the light gets partly scattered and partly transmitted directly. If the amount of scattering is sufficiently low (as in the top row of Figure 8) or the object is sufficiently thin (as in the bottom row of Figure 10), the background is visible through a translucent object. In this case, the existing transparency models might, to some extent, contribute to the explanation of perceived translucency.

**Figure 1.**
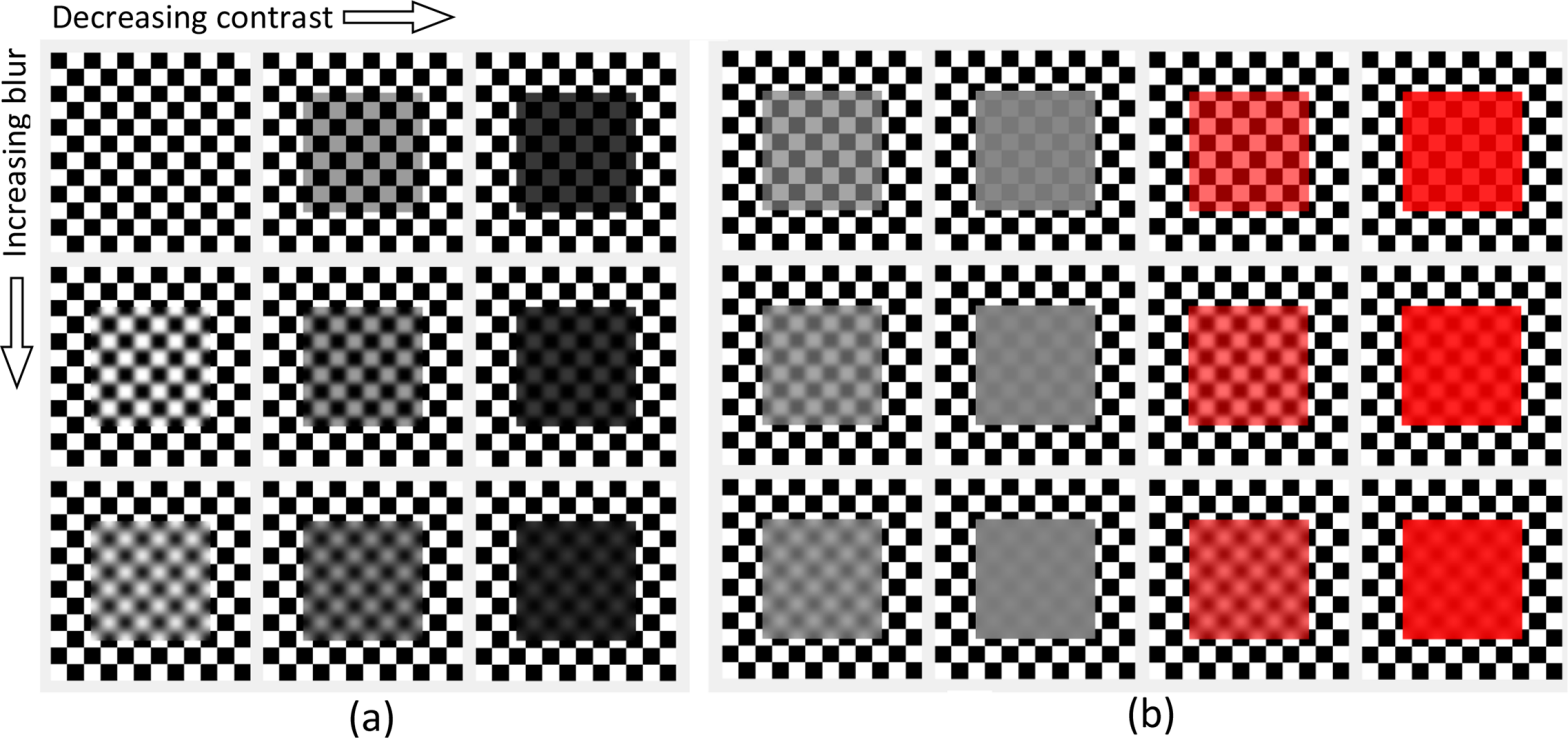
(a) A vivid impression of transparency and translucency has been produced by simple image manipulations. The contrast was reduced by decreasing lightness of the white patches of the checkerboard that yields an impression of an absorbing transparent filter with no reflection. Application of the Gaussian blur generated translucent look for all levels of contrast. Top row - no blur; Middle row — σ=12; Bottom row — σ=20. (b) In the left two columns, the contrast is decreased by decreasing the lightness of the white patches and increasing the lightness of the black patches, as if the filter had direct reflection. A convincing translucent appearance is generated, even when no blur is applied (top row). Translucency is stronger and more convincing with reflectance (additive) component than it was for the absorption-only scenario (in the top row, compare the rightmost image in (a), and the second one from the left in (b)). The two rightmost columns demonstrate the chromatic case, where the hue shift also produces a hazy look and contributes to translucency appearance.

Internal scattering affects the clarity of the background image. Blur of the see-through image produces a translucent look (refer to Figure 1 and also Figure 19 in Singh & Anderson, 2002b). It has been demonstrated that a change in the internal scattering produces a larger apparent translucency difference when the background is visible and blurred, than it does for highly scattering materials (Gigilashvili et al., n.d.). Singh and Anderson (2002a) extended transparency research to thin see-through filters that scatter light. Scattering blurs the image and usually decreases the contrast. In most cases, the two parameters covary. The authors demonstrated that the blur alone decreases perceived transmittance when the Michelson contrast is fixed (Michelson contrast is defined as (I_max_ − I_min_)/(I_max_ + I_min_), where I_max_ and I_min_ are the maximum and minimum luminances, respectively, Legge et al., 1990). Although they also found that the apparent contrast is smaller owing to blur even if the Michelson contrast is kept constant, the decrease in perceived transmittance cannot be fully attributed to that. They propose that both blur and contrast of the transmitted image are the cues that increase the perception of opacity and decrease perceived translucency. A similar observation was made by Gigilashvili et al. (2018a), who studied blur from the image quality point of view and found that blurring removes the transmission cues and impairs translucency perception.

Visibility of the background through a medium is indicative of the subsurface light transport and can inform the HVS about translucency (e.g., see Figures 5, 8, and 10.). Seeing through a medium has been broadly studied in the context of transparency. The visual stimulus reaching a human retina through a transmissive material is a mixture of the contributions by the background and the transparent overlay. The HVS perceives the background as a single surface, even though the colors of the background in a plain view and those seen through a transparent medium might differ considerably. We somehow understand and estimate the properties of a transparent medium superimposed on a background. To infer transparency and distinguish transparent substances from opaque ones, the HVS relies on the regularities that exist between the colors of the background in a plain view and those seen through a transparent medium. Transparency is perceived when the lightness and chromatic compatibility exists between the overlay and the background. Modelling transparency perception has developed in two primary directions. Some works model transparency in a form of an *additive color mixture* (Metelli, 1970; Singh & Anderson, 2002b). An example of the additive model is the episcotister model by Metelli (1970, 1974, 1985). The idea of the episcotister is the following: a disc with a sector cut out is rotating with high speed and is seen as a transparent overlay over an opaque background. The colors of the disc and the background simply add algebraically, and the proportions depend on the angle of the cut-out sector. Although colors are mixed over time in Metelli's model, additions can happen spatially as well — for instance, an opaque mesh with small holes looks partly transmissive as a whole (Singh, 2020). The same principle has been later extended to the chromatic cases as well (D’Zmura et al., 1997; Hagedorn & D’Zmura, 2000). D’Zmura et al. (1997) studied the relation between colors at the background-overlay junctions and found that a shift in colors and change of the contrast are responsible for transparency perception. For instance, if the colors either converge toward a point or are translated in the color space, they induce the percept of transparency, while rotations and shear do not lead to the same effect. Additive models approximate well the phenomena such as fog (Hagedorn & D’Zmura, 2000) or the media shown in the top row of Figure 8.

However, many transparent materials we encounter on a daily basis, such as glass, plastic, or beverages, involve more complex optical phenomena. The transparency of the media similar to those shown in the bottom row of Figure 8 can be described with the *filter models*, which involve a *subtractive color mixing*. The filter models have been proposed both for the achromatic (Beck et al., 1984) as well as chromatic stimuli (Faul & Ekroll, 2002, 2011; Khang & Zaidi, 2002). This approach models the transparent overlay as an optical filter, which absorbs part of the light propagating through it, but also reflects some of the incident illumination at the vacuum-filter interface as per Fresnel equations. The color seen through the filter is a combination of the transmitted and reflected components.

There are two primary reasons why considering transparency perception models are also important for translucency.

First, it has been demonstrated that if particular regularities between background and transparent overlay colors are absent (D’Zmura et al., 1997; Faul & Ekroll, 2002), the filter is perceived opaque. Therefore, we believe that that kind of compatibility between the filter and background colors is also significant for translucency perception (it is worth noting that similar kind of chromatic compatibility is needed for gloss perception as well, Nishida et al., 2008). The future work should reveal to what extent is the perception of translucency dependent on these regularities and whether translucency can be perceived in the cases when the filter and background colors are incompatible for inducing transparency perception (e.g., assuming fluorescence).

Second, a vivid perception of translucency can be evoked by transparent filters even in the absence of blur (i.e., if the contours in the background image remain undistorted). This means that, when the background is visible, translucency can be observed even without any internal scattering. This can be ascribed to the decreased contrast and the color shift in the see-through image (Figure 1). If the transparent filter absorbs (subtractive color mixing) or reflects light (additive component), the contrast in the see-through image is decreased. Human observers are usually able to identify the additive component as a mirror reflection of the environment. Hence, the reflections from the surface usually evoke perception of gloss (as in the bottom row of Figure 8). However, Faul and Ekroll (2011) have demonstrated that specular reflections under uniform diffuse illumination evoke perception of translucency instead of gloss, proposedly because surface scattering is mistaken for volume scattering (see Figure 1). They also extended their prior work on filter models (Faul & Ekroll, 2002) and proposed an alternative parametrization of filter's physical properties — thickness, absorption and refractive index. They propose hue (H), saturation (S), transmittance (V), and clarity (C), to quantify the perceptual dimensions of transparency. The dimensions are related to the physical parameters; for instance, transmittance decreases exponentially with the filter thickness, and clarity is related to the index of refraction. Although the model does not account for subsurface scattering, V and C yield a broad range of appearances across the transparency-opacity continuum. The index of refraction determines the amount of the direct reflection from the surface. If it is equal to the refractive index of the immersing medium, no light is reflected at the interface, yielding the maximum clarity. However, a high reflection from the surface yields hazy translucent appearance (see Figure 1). A more perceptually uniform version of this space has been recently proposed by Faul (2017). The author made another interesting observation: the filter reflections and the resulting lack of clarity induce the perception of transparency and translucency when the luminance contrast in the background is large. However, the effect becomes weaker on low-contrast backgrounds. For instance, if a homogeneous background was used instead of a checkerboard, the filters shown in Figure 1 would have appeared uniform opaque patches. Faul (2017) proposes motion as one of the factors for disambiguating this kind of stimuli. This and other factors contributing to apparent translucency or facilitating perception of translucency is discussed in Factors impacting translucency.

## Factors impacting translucency

Translucency as a visual attribute is impacted by different intrinsic and extrinsic factors. We provide an overview of the knowledge status on them.

### Intrinsic parameters

#### Absorption and scattering coefficients

**Figure 2.**
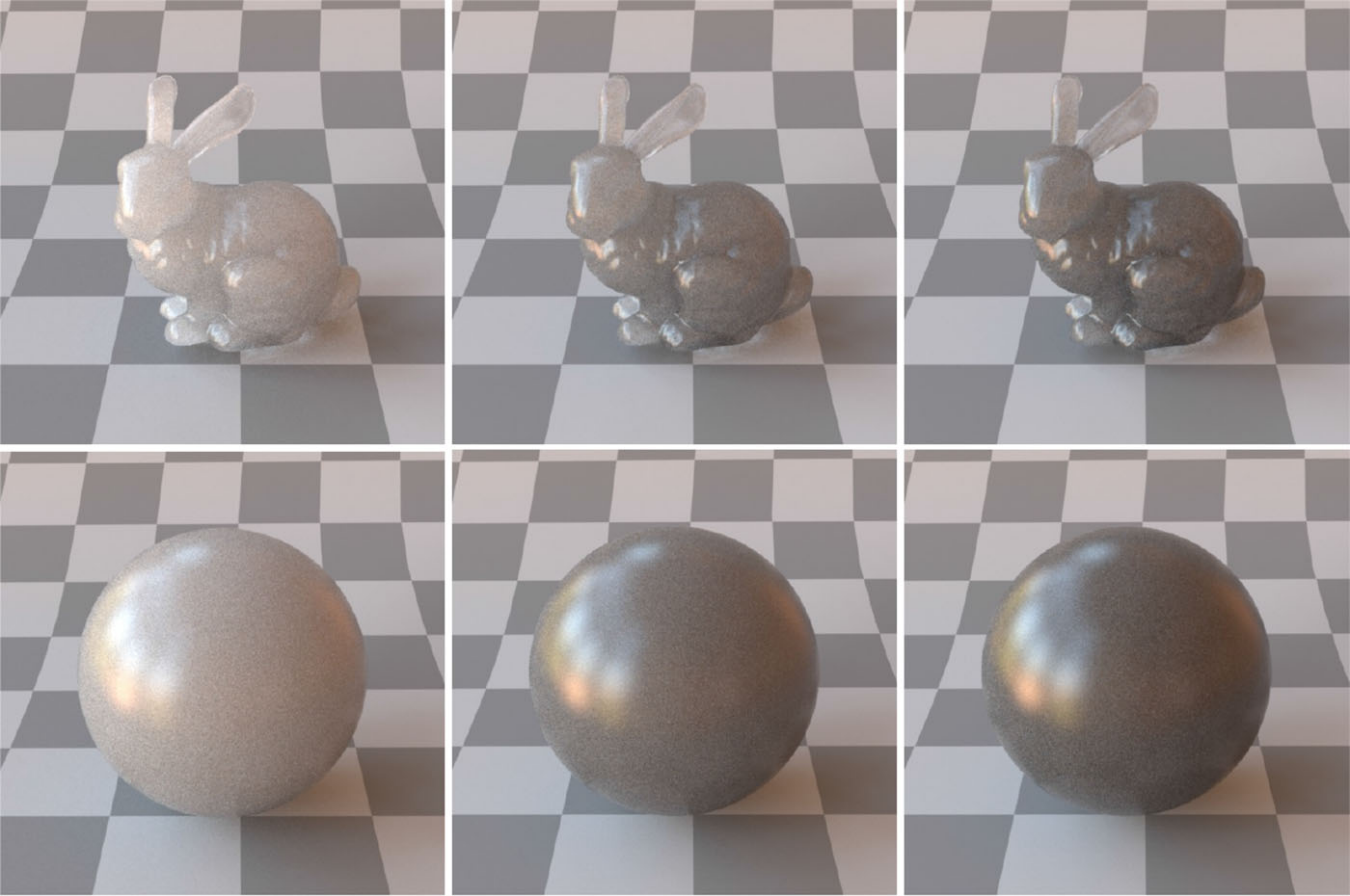
Objects in the same column are made of the identical material. However, due to smaller scale and presence of thin parts, the Bunny has more cues evoking perception of translucency. Objects in the first column have high scattering and low absorption. In the second column, lower scattering and higher absorption. In the third column, the same scattering as in the second column, but higher absorption. How can we compare their perceptual translucency? Which of these six objects or materials are the most and the least translucent? (Reproduced from Gigilashvili et al., 2020b).

Wavelength-dependent absorption and scattering coefficients are fundamental parameters that describe the radiative transfer through a medium. Scattering (σ_s_) and absorption (σ_a_) coefficients signify the scattering and absorption events per unit distance traveled by a photon, respectively. The sum of the absorption and scattering coefficients is called extinction or attenuation coefficient (σ_t_). The extinction coefficient σ_t_ is given as a sum of the scattering and absorption coefficients (σ_s_+σ_a_, respectively). The σ_t_ for perfectly transparent material is equal to zero. A high σ_a_ means that fewer photons escape the material and the object gets a darker shade; per contra, a high σ_s_ is responsible for blurry and shiny appearance. It is worth mentioning that in addition to volume scattering (scattering inside the medium), a scattering event can also take place at the surface (as discussed elsewhere in this article). Xiao et al. (2014) demonstrated that the increase in the optical density of the materials affects translucent material matching in a monotonous and linear way under all illumination geometries. The effect of different absorption and scattering coefficients is shown in Figure 2.

**Figure 3.**
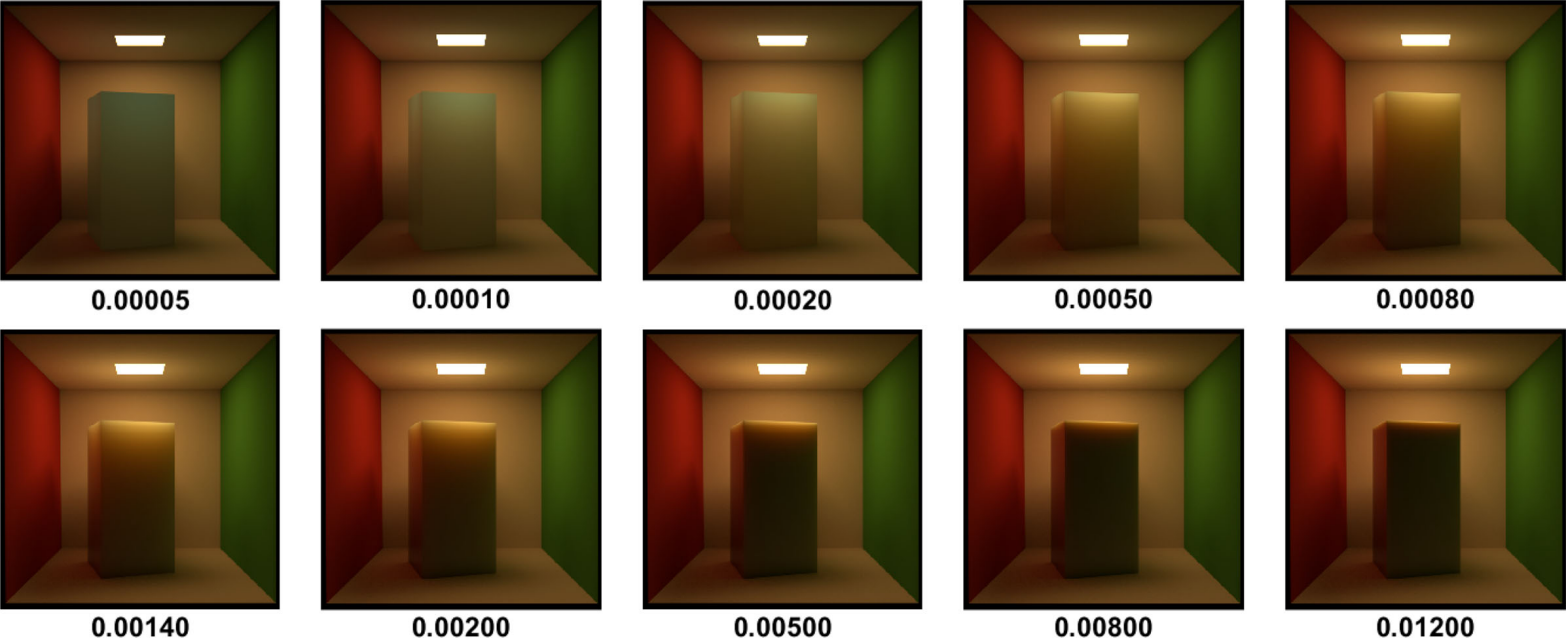
We rendered the box with skimmed milk optical properties as measured by Jensen et al. (2001) and implemented in Mitsuba (Jakob, 2010) in a Cornell Box (Niedenthal, 2002) (a broader variety of measured scattering properties can be found in the work by Narasimhan et al., 2006). The optical density was varied with a scale parameter (shown below the image). It is apparent that the penetration depth decreases monotonically with the optical density. Therefore, only the edges are bright in the optically thick materials and the contrast with the rest of the object is large. On the other hand, photons spread easily through optically thin materials yielding relatively homogeneous luminance distribution.

**Figure 4.**
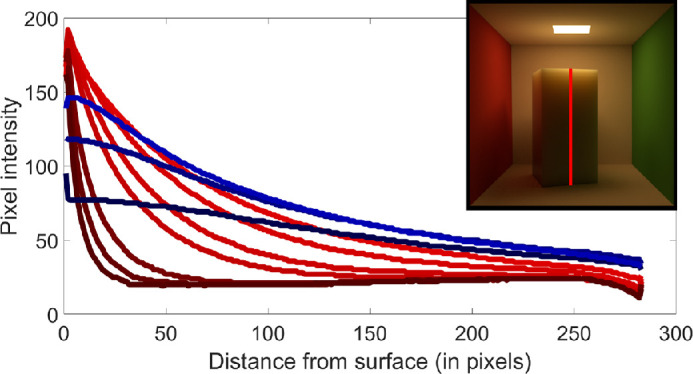
Image intensity as a function of the distance from the incidence surface. The cross-section where the intensities are measured is marked with a red strip in the top right corner. Optically thicker materials are shown in red (darker the shade, denser the material). The intensities are high at the boundary and they increase in the near vicinity, reaching local maxima - as proposed by Gkioulekas et al. (2015) (as discussed elsewhere in this article), then they monotonously decrease as the depth increases. Optically thin materials are shown in blue (a lighter shade corresponds with a thinner material), because they behave differently. They do not have a high intensity near the edge and the decrease slope is smaller. This supports the proposal by Koenderink and van Doorn (2001).

Cunningham et al. (2007) studied aesthetic correlates of physical attributes and found that absorption and scattering are embedded onto a one-dimensional manifold where they are significantly correlated with the semantic labels of “brightness” and “blackness.” Koenderink and van Doorn (2001) illustrated that materials with high mean free path look relatively uniformly shaded as the photons propagate through the material easily. In contrast, if the mean free path is short, the penetration depth is shorter (Motoyoshi, 2010) and the radiant energy is visible near the edges on the side of the incident beam, while the rest remains relatively dark. This is illustrated in Figure 3. How intensity varies as a function of the distance from the surface, is illustrated in Figure 4.

Chadwick et al. (2018) demonstrate that although imperfectly, human observers are still able to unmix absorption and scattering in milky tea images. They tried to identify potential image cues used by observers and found that mean saturation explains well the variation in observer responses on the milkiness estimation task (which is accounted for scattering). In contrast, value (V of the HSV) and the spatial saturation gradient were needed to explain the tea strength (absorption) responses. Interestingly, the cross-individual variation was large; different observers seemingly rely on different perceptual functions or simply interpret the concepts differently. Urban et al. (2019) proposed a perceptually uniform translucency metric, which encapsulates the observation that the HVS is more sensitive to absorption-scattering differences in optically thin materials than in optically thick ones. The same was observed by Gigilashvili et al. (2019c, n.d.). They found that, if a material is nearly transparent, even a slight change in absorption and scattering coefficients is easily detected by humans, whereas larger steps are needed to notice the difference in more opaque materials.

Vu et al. (2016) observed that, for textureless, flat thin 3D-printed shapes, transmittance is more perceptually important than lateral light transport. They quantified the ratio of transparent and scattering white material in the mixture on a 255-level *gamma* scale, where low gamma corresponds to a higher portion of the scattering colorant and found that within the range of 0 to 180, that is, more than 70% of the physical parameter-space, transmittance was negligibly small (and perceptually opaque), whereas in the remaining range human observers were sensitive to colorant ratios, as the transmittance and the perceptual correlate were well-explained with the Stevens’ power law (Stevens, 1960).

Despite those attempts, the question on how exactly absorption and scattering coefficients contribute to perceptual correlate of translucency remains largely unresolved. One of the problems is that the perceptual dimensions of translucency are not known and the relation with transparency and opacity remains fuzzy. One of the recent attempts to structure translucency in a physical parameter space was made by Gerardin et al. (2019). They proposed a 3-D translucency classification space for computer graphics — a cube where dimensions correspond to absorption, scattering and surface roughness. They claim that, by increasing scattering, a transparent material gradually becomes translucent and then eventually opaque. However, by increasing absorption, a transparent material gradually becomes opaque, but never translucent.

Finally, the amount of the radiant energy that emerges from an object can be result of not only subsurface scattering (or surface reflection), but also emission (Tominaga et al., 2017). To the best of our knowledge, no study has investigated translucency perception on fluorescent materials. How well the HVS can separate the light emerging from a material into transmitted and emitted components, or whether we can tell the difference between translucent and fluorescent stimuli should be answered in the future.

#### Scattering phase function

**Figure 5.**
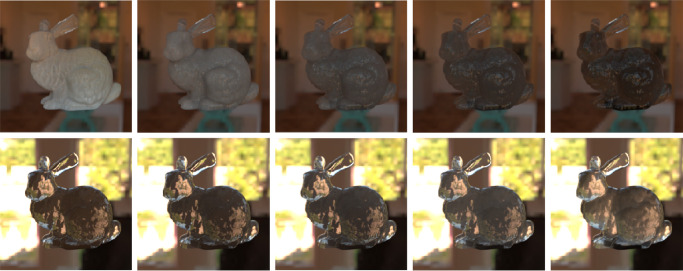
The images vary in the phase function, while all other intrinsic properties are kept constant. Single lobe (Henyey & Greenstein, 1941) phase function is used with a varying value of *g*. The parameter *g*, is usually defined in the range of [−1 1], where negative values imply backwards scattering (back to the direction the light is incident from), positive values mean scattering forward, and 0 corresponds with the isotropic scattering. In the columns left to right *g* is equal to −0.9, −0.5, 0 (isotropic), 0.5 and 0.9, respectively. The top row is rendered in the front-lit illumination geometry, and the bottom row is back-lit. Because of the low optical density of the material, the direct transmission is high in the back-lit condition and the impact of the scattering directionality is negligible. The opposite is true for the front-lit condition. In case of back-scattering, more photons are redirected towards the camera, while the forward-scattering phase function redirects photons away from the camera. The appearance varies strikingly and ranges from almost Lambertian diffusive (owing to high backwards scatter near the surface) to blurrier translucent looking (*g* = −0.5 and isotropic) and to darker, opaque-looking one. Please note that in case of forward scattering, thicker parts of the bunny look more opaque, and thinner parts look translucent, as the forward scattering phase function facilitates transmission from the background toward the camera.

Although the likelihood and the number of scattering events are essential, the direction a scattered photon is redirected to can also be important. If multiple scattering is assumed (Jensen et al., 2001), where diffuse approximation can be applicable, the impact might not be that strong. However, it can have a striking impact on the thin parts of the object, where only few scattering events take place (although in some cases, a phase function can impact thick parts too; refer to Figure 5).

Gkioulekas et al. (2013) have conducted a comprehensive study on the role of a phase function in translucent appearance. They argue that a similar translucent appearance can be yielded with the contrasting phase functions and conclude that a perceptual translucency space is composed of a lower number of dimensions than the physical parameter space. They generated a broad range of phase functions by linearly combining multiple Henyey-Greenstein and von Mises-Fisher lobes. Afterward, they conducted psychophysical experiments and came up with a two-dimensional perceptual space of phase functions, where each dimension modulates diffusion (i.e., milky appearance) and sharpness (i.e., glassy appearance), respectively. The contribution is significant for material design and has expanded the gamut of possible translucency, because many of the appearances would not have been reproducible with a single lobe phase function. However, the robustness of the space is partially compromised in back-lit illumination geometry. Xiao et al. (2014) have extended the work and found that, although the illumination direction usually affects the perceived magnitude of translucency, this impact is not significant for some phase functions. They found that phase function's location in the perceptual space (which was proposed by Gkioulekas et al., 2013) defines whether an illumination direction impacts perceived translucency. The similar correlation has been found between a phase function and translucency constancy (Xiao et al., 2014). The general trend is that the impact of lighting directionality is stronger for phase functions producing sharp glassy results than for more diffusing ones, which is intuitive; nearly isotropic phase functions that scatter light in all directions will be less affected than the ones that redirect photos strictly towards particular directions. Although Xiao et al. (2014) argue that the role of the phase function is also dependant on the object shape, the exact covariance between the shape and the impact of the phase function needs to be addressed in more detail.

**Figure 6.**
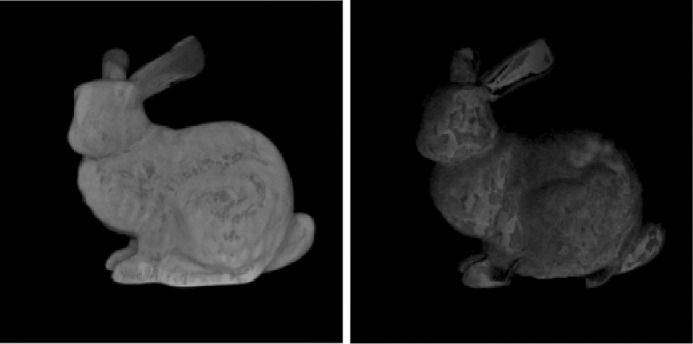
The intensity difference between the two extremes of the forward and backward scattering lobes in front-lit (left) and back-lit (right) illumination conditions. In the the front-lit condition, the difference is striking, while it is less apparent for the back-lit illumination condition. This difference can be attributed to the fact that, owing to low optical density of the material, direct transmission is high when illuminated from back and the scattered light accounts for a smaller portion of the resulting appearance.

Figure 5 illustrates a simple case of how the phase function alone can impact appearance, while all other parameters remain fixed. The images are rendered with a single lobe (Henyey & Greenstein, 1941) phase function, which takes a parameter *g* to define the directionality of the scattering. In the front-lit illumination geometry (top row), backward scattering resulted in brighter and more diffuse look, as the photons were scattered back toward the camera. On the contrary, forward scattering redirects photons away from the camera, resulting in dark opaque-looking appearance (although, note that thin parts look see-through, because the background reflections are forward-scattered *toward* the camera). In contrast, the impact is negligible for the back-lit illumination condition (bottom row), because strong directional backlight results in high direct transmission and the magnitude of scattering change has weak impact on the resulting appearance. Figure 6 illustrates that the difference between the two extreme cases of the phase functions is striking for front-lit conditions (left image), whereas it remains subtle for back-lit conditions (right image).

#### Index of refraction

**Figure 7.**
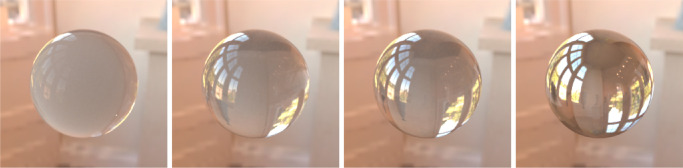
The only optical property that varies among the four images in the index of refraction (1.10, 1.33 [water], 1.50 [glass], and 2.41 [diamond], from left to right, respectively). A low refractive index ends in the lower Fresnel reflection and higher portion of the light penetrating the subsurface. Therefore, scattering in the subsurface is more apparent and the leftmost image looks more translucent. In contrast, a high refractive index leads to higher reflection ratio and lower transmission, which yields glossy specular appearance rather than translucent one (refer to the rightmost image).

**Figure 8.**
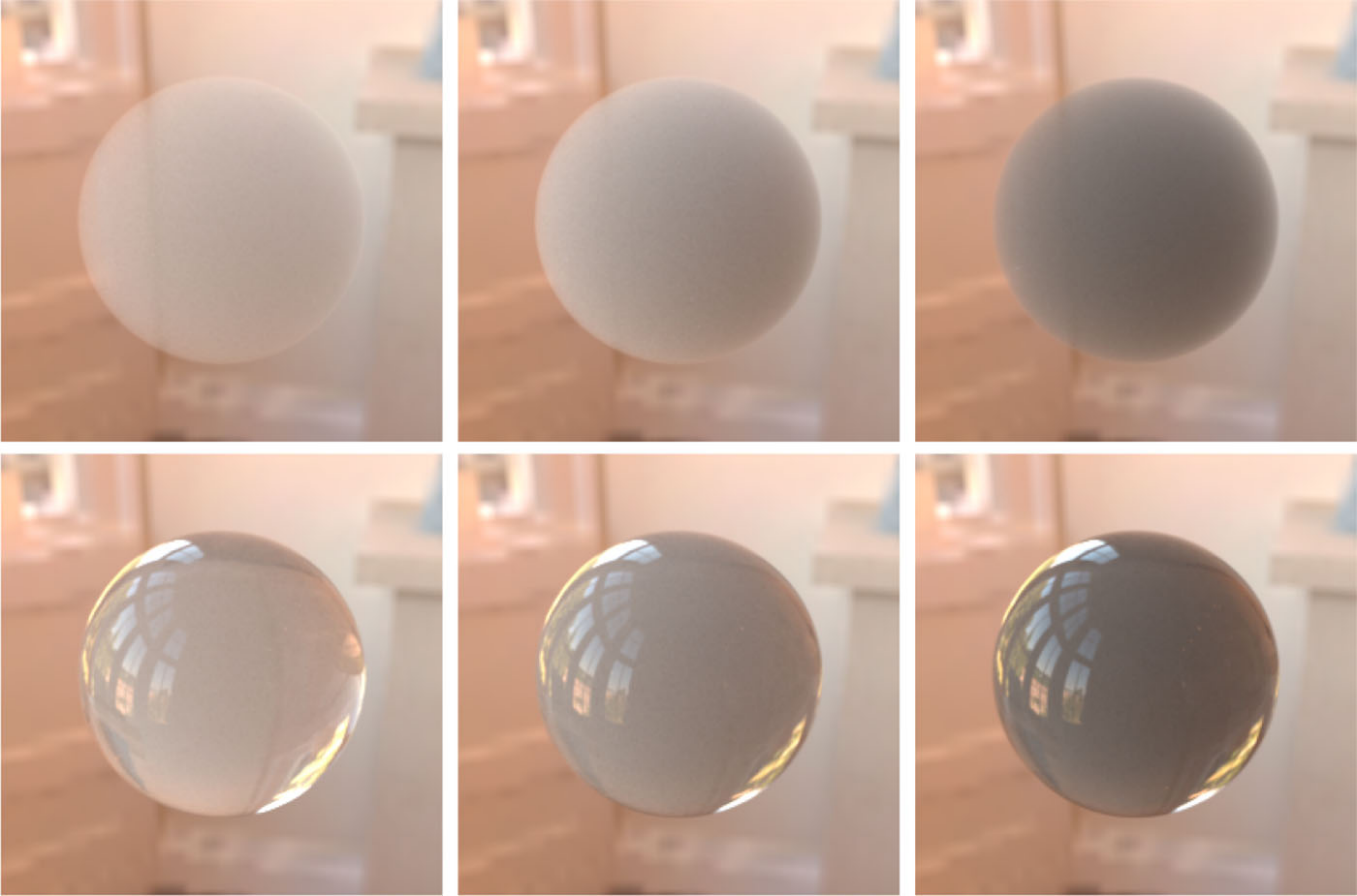
Glossiness is not essential for sensation of translucency. In the top row, the difference between the ambient vacuum and the object refractive indices is negligible, which results in nearly no refraction and, thus, no specular reflections. Despite the absence of the glossiness cues, the object still seems to be translucent, but the material looks more like smoke or a sponge. In the bottom row, specular reflections are added, and the scattering properties inside the participating medium is identical to those of the top row. The material looks more glassy and more realistic, because the bottom row objects are more likely to be encountered in the real life than their top row counterparts. However, we cannot comment on whether glossiness actually increases perceived magnitude of translucence.

The index of refraction is one of the most understudied intrinsic material properties in the context of translucency perception. At the boundary of the media, the difference between their refractive indices defines the angle the light ray is refracted with. Therefore, the refractive index has a strong impact on the background distortion in see-through images (proposedly also contributing to shape perception Schlüter & Faul, 2019). Fleming et al. (2011) have shown that humans are surprisingly good at estimating refractive indices of transparent materials, proposedly relying on a background distortion cue (although subject to biases owing to the object's thickness and distance to the background). Afterward, Schlüter and Faul (2014) argued that instead of estimating an abstract refractive index, the HVS rather performs image-based matching where the both background distortion and the specular reflections are contributing. Regardless of these attempts, the role of the refractive index in the appearance of non-see-through materials remains understudied. Additionally, difference in the refractive indices of the two bounding media modulates the magnitude of the Fresnel reflection and transmission, more refractive objects usually appearing glossier (Fleming et al., 2011; Schlüter & Faul, 2019) (also impacting caustics; Kán & Kaufmann, 2012; Lynch et al., 2001). This is illustrated in Figure 7. Although the subsurface scattering properties of a material remain constant, a high refractive index can render a mirror-like look and decrease perceived translucency (which is rooted in the decreased Fresnel transmission). If the difference between the refractive indices of the bounding media is negligible, hardly any specular reflections are generated and a smokey-looking participating medium appears (see the top row in Figure 8 and compare with the bottom row in the same figure).

**Figure 9.**
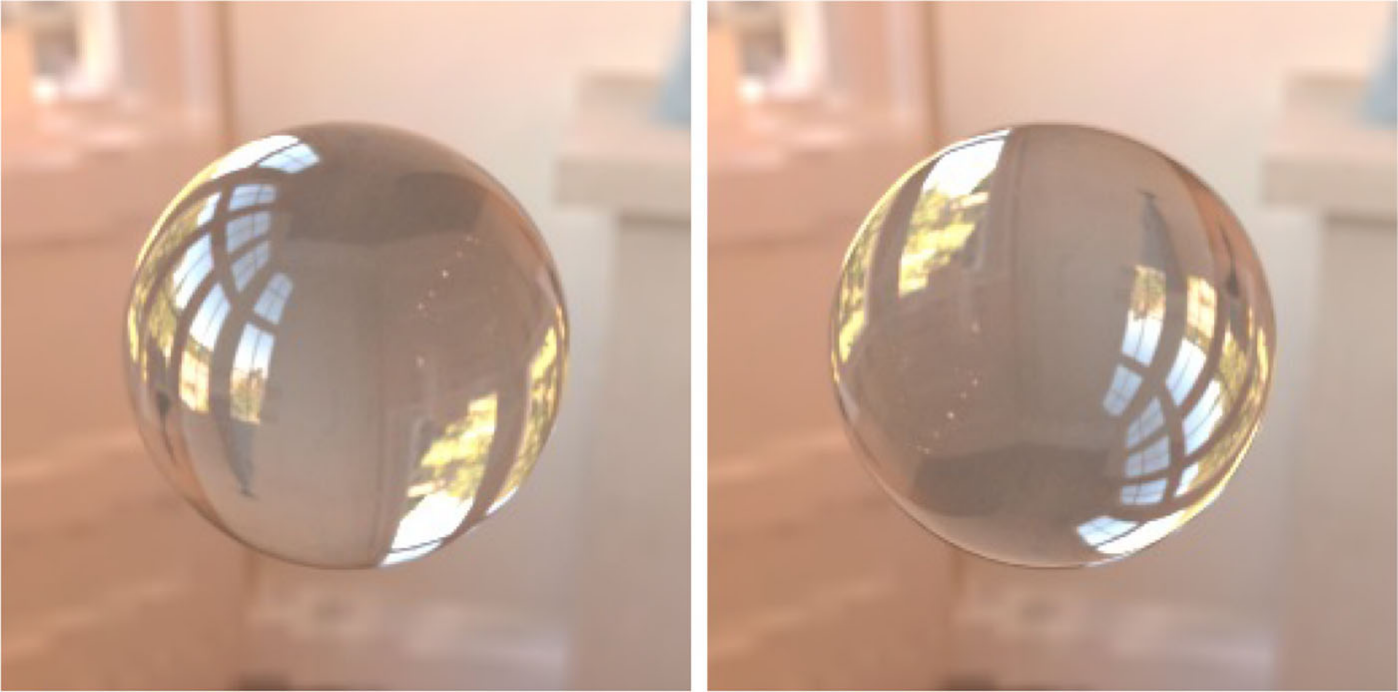
The transmission image in the left photograph is upside-down, which indicates that it is the result of the refraction through a convex lens. If we simply rotate the sphere upside-down, then the transmission image will look more like an opaque mirror reflection. This was first demonstrated by Kim and Marlow (2016).

Observers’ knowledge of the geometrical optics and the refraction phenomenon can facilitate distinction between the transparent media and mirror-like reflectors. Although the convex lens refracts the light and transmission image is superimposed on the object upside down, the convex mirror reflects the environment upright. Kim and Marlow (2016) have observed that rotating an image of a transparent sphere upside down creates an illusion of reflection, instead of transmission. This effect is illustrated in Figure 9.

The refractive index also determines the internal reflections (when the light is reflected backward when it is trying to leave the translucent material), which impacts the amount of radiant energy emerging from the material — thus, also translucency cues. The extreme case is the total internal reflection — when the light traveling from a medium with a higher refractive index is fully reflected backward — thus, no refraction happens and no light emerges from that medium to the medium with lower refractive index. The total internal reflection takes place when the angle of incidence is larger than the critical angle. Therefore, it is more likely to happen on complex surface geometries, rather than smoother ones. This could be one of the reasons for the appearance difference between the smooth and the complex Lucy shapes in Figure 13.

Marlow and Anderson (2021) have shown that, if the illumination and observation angles nearly match, refraction can affect translucency, because the portion of the light exiting the material is reflected internally and is redirected toward the convex and away from the concave regions. The effect is relatively weaker when the difference between the indices of refraction of the bounding media is low and nonexistent when the difference between the observation and illumination angles is large.

Finally, **polarization** of the incident light can also play a role in the Fresnel reflection and transmission. Gkioulekas et al. (2015) have used cross-polarization photography to remove undesired specular reflections. They argue that specular reflections affect the location of the maxima and compromise the robustness of their radiance edge profiles for translucency prediction (to be discussed later). However, it might not be important for rough surfaces. Polarization is a broadly unexplored extrinsic property that deserves attention in translucency perception research.

### Extrinsic factors

#### Object scale and structural thickness

**Figure 10.**
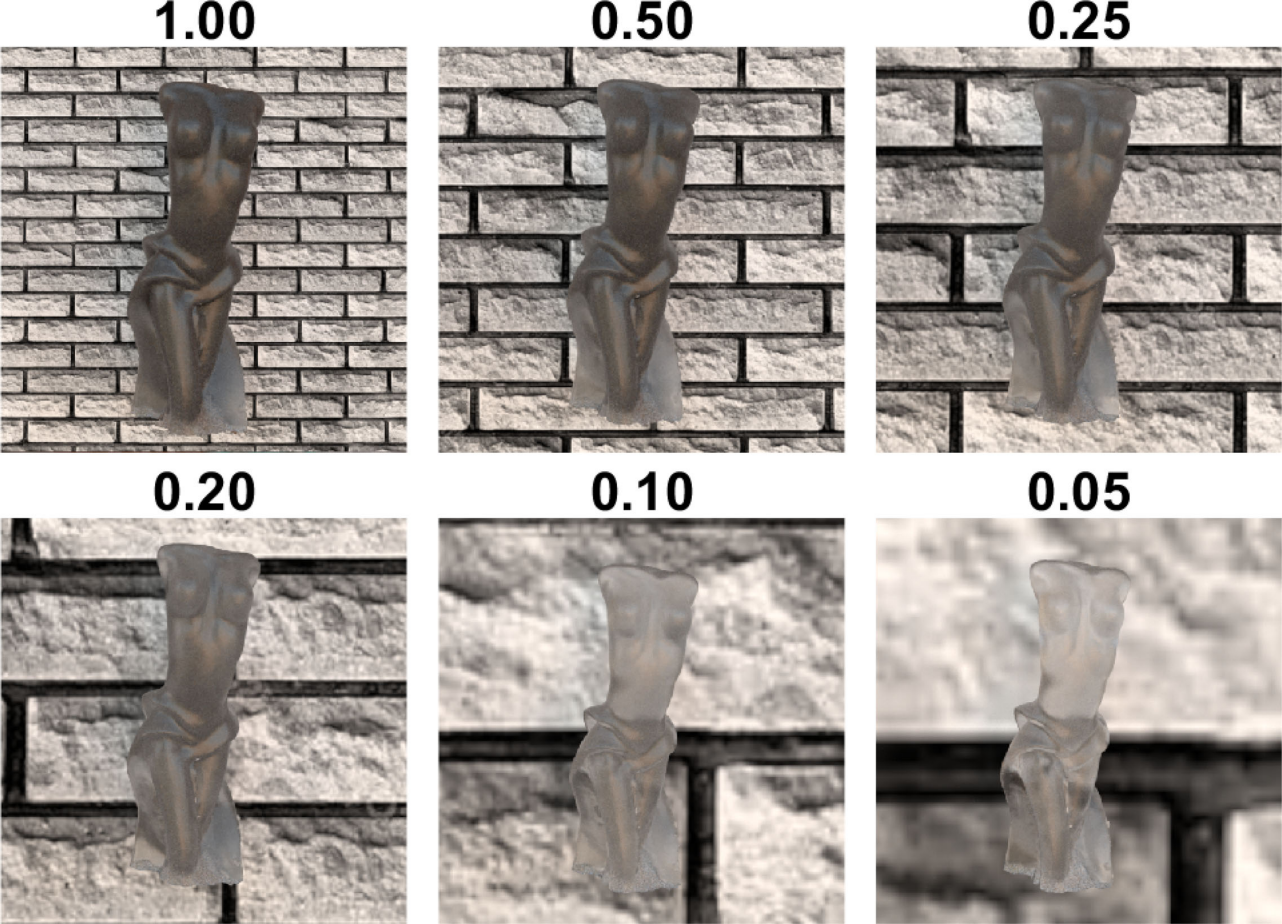
The illustration of how the object scale impacts perceived translucency of an object. Although all six figures differ in scale, they have an identical shape and are made of an identical material. The smaller scale of an object means that a photon needs to travel a shorter distance to go through the material; that is, for given scattering and absorption coefficients, the likelihood of scattering and absorption events decreases. This makes larger objects look more opaque and smaller ones look more light-transmissive. The numbers correspond with the scale relative to the top left object. The background texture can also facilitate understanding the scale differences. We also illustrate that when the object scale varies, perceived translucency is also strongly impacted by the resolution of the image. If we put these six figures in a single scene, side by side (e.g., if we put the 0.05 version next to the original one in the 1.00 scene), smaller ones might look opaque, because the luminance variation will not be detected owing to the contrast sensitivity limitations.

If the object is enlarged, the distance a photon needs to travel increases. This means that, for a given extinction coefficient, the number of absorption and scattering events goes up and fewer photons escape the material unscattered. The opposite is true, if the object is smaller. Therefore, object scale has an impact on the translucent appearance (Fleming & Bülthoff, 2005). This has serious consequences for 3D printing. Urban et al. (2019) have proposed *Alpha* — a psychophysics-based perceptually uniform translucency metric. However, the authors highlight that the metric should be scaled with the object size and provide a proper implementation of this. How object scale impacts appearance for a fixed optical material properties is illustrated in Figure 10 (also compare Bunny with a sphere in Figure 2). Photons need to travel a shorter distance at the edges — making them bright and thus, a characteristic cue for distinguishing translucent and opaque materials (Fleming & Bülthoff, 2005; Gkioulekas et al., 2015). Gkioulekas et al. (2015) have observed that the radiance profile at the edges are surprisingly robust and invariant toward illumination changes, making them a reliable “signature” for a material translucence.

**Figure 11.**
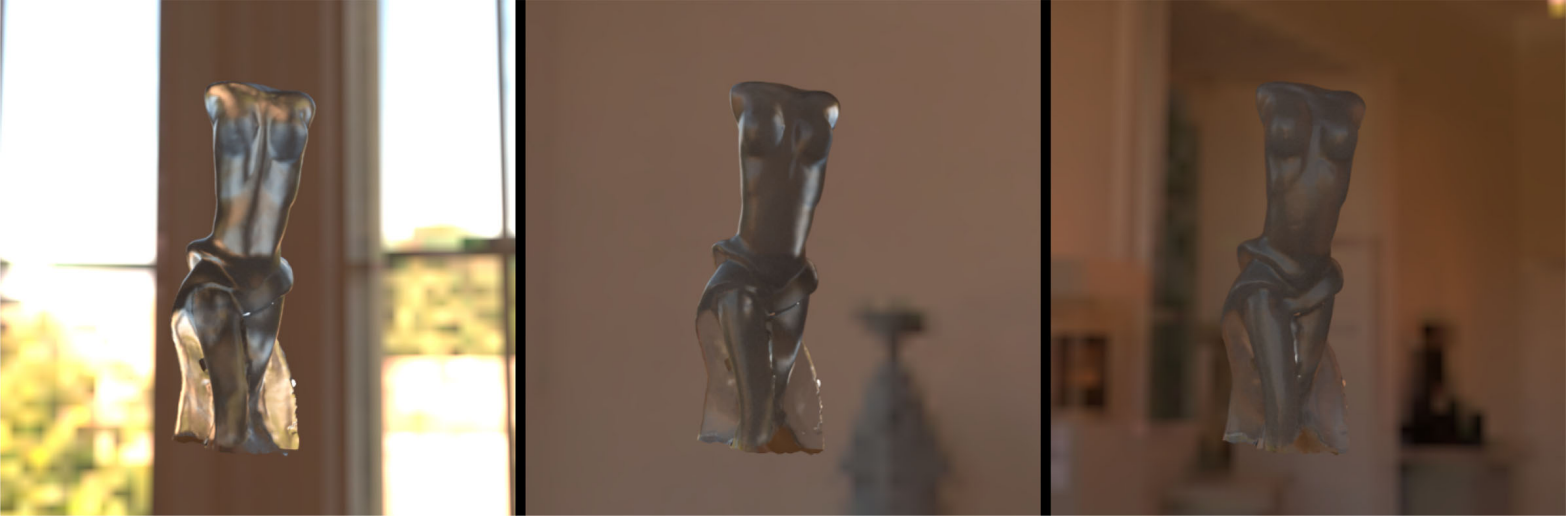
The three frames are taken from a video. Refer to Supplementary Material 1 for the video. The object is identical; however, the illumination geometry varies from back-lit (left) to side-lit (middle) and front-lit (right). The video provides a vivid illustration of how the perceived translucency changes with the change of the illumination directions. Moreover, it demonstrates that motion facilitates perceiving material translucence. Finally, the object shape enables us to observe how the presence of the thin parts provides additional cues about the light transmission properties of a material.

Depending on the structural thickness, the translucency appearance of a given object made of a homogeneous material can vary considerably. Refer to the Figure 11. Although the torso of the bust usually looks darker and less see-through, the thin parts of the dress transmit more light in all illumination conditions and look especially shiny when back-lit. The same is true for the ears of the Bunny (Figure 5). It has been shown that presence of the thin parts can facilitate detection of translucency differences (Gigilashvili et al., 2019c, n.d.), proposedly attributed to the fact that the HVS is more sensitive toward the changes in optically thin materials (Urban et al., 2019). This is further substantiated by Sawayama et al. (2019), who propose that a rugged surface of the object facilitates discrimination of translucency. Both findings indicate that the parts where a photon needs to travel the shortest distance contain the most information about material translucence. Also, materials with a heterogeneous structural thickness might overall look more translucent and less opaque when they have thin parts. This is true both for solid objects (Gigilashvili et al., 2018b, 2021b), as well as liquids (see the role of wavetips in sea paintings; Wijntjes et al., 2020).

#### Surface roughness and geometry

**Figure 12.**
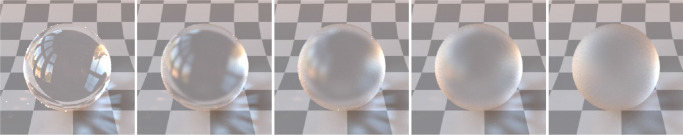
In addition to the subsurface scattering, surface scattering also blurs the background and generates translucent appearance. The sharpness of the specular highlights provide a strong cue for estimating surface scattering (Pellacini et al., 2000; Thomas et al., 2017). However, when the surface scattering is high, estimating subsurface scattering properties becomes increasingly difficult (e.g. see the right image: can we tell whether a subsurface is composed of a transparent or scattering material?). The root mean square slope of microfacets equals to 0, 0.05, 0.10, 0.15, and 0.25, from left to right, respectively.

Micro- and macro-scale surface geometry, although both scatter light, have qualitatively different effects on appearance. The microfacet-level surface roughness impacts refraction (Xiao et al., 2014), blurs the background image and evokes the perception of translucency, even for the materials with zero subsurface absorption and scattering (Gigilashvili et al., 2020a). It has been observed to be positively and monotonously correlated with translucency, when the transparency is seen as the other extreme (Gigilashvili et al., 2020a). In the translucency classification system for computer graphics, proposed by Gerardin et al. (2019), surface roughness is one of the fundamental dimensions in the 3-D parameter space. The authors argue that an increase in surface roughness makes a transparent object translucent, but never opaque; regardless the roughness of the surface, some photons still manage to go through (if the material has large mean free path). This phenomenon is shown in Figure 12.

**Figure 13.**
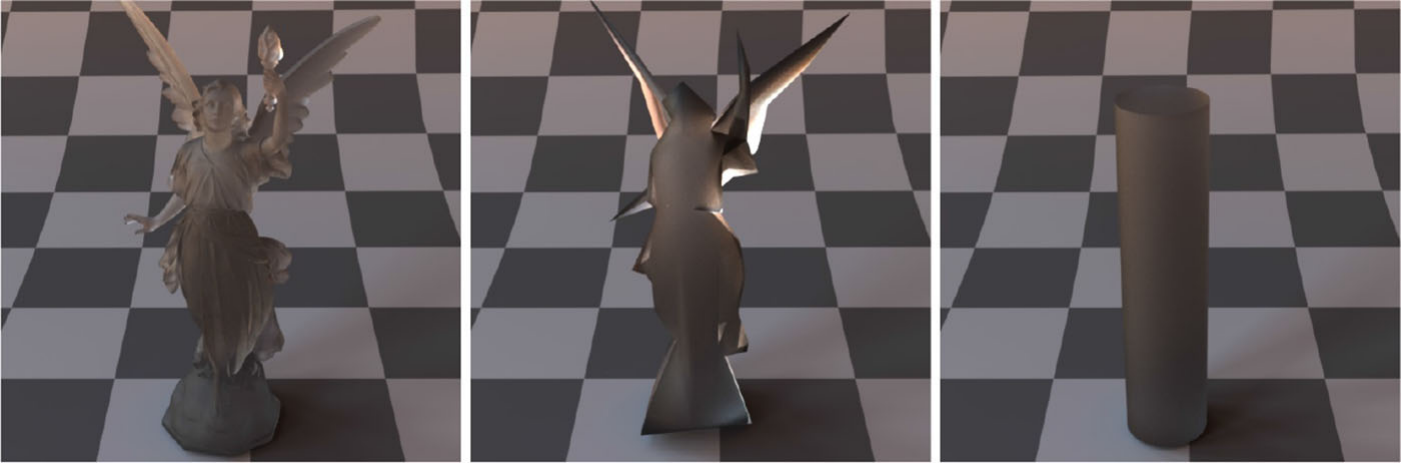
All three objects are made of the identical material. The Lucy (left) has the sharpest edges, while the sharpness and the surface curvature decreases gradually for low resolution Lucy (middle) and a cylinder (right). However, it is difficult to speculate which one yields the most vivid perception of translucency.

According to the literature, translucency can impact perceived macro-scale surface geometry of the object — translucent objects appearing less sharp (Chowdhury et al., 2017). Interestingly, Xiao et al. (2020) have found the correlation the other way round too — experimenting with different levels of surface relief and claiming that presence of sharp edges make materials appear less translucent. They partially attribute this to the local contrast generated by the shadows owing to high surface reliefs. However, the surface relief on a relatively flat surface is a tiny subset of the potential surface geometries which yield sharp edges. For instance, refer to Figure 13. The Lucy (on the left) has the sharpest edges and the most fine details; the low-resolution Lucy (Gigilashvili et al., 2021a) (a smoother version of Lucy with a smaller number of vertices) has fewer and less sharp edges, whereas the cylinder is the least sharp among the three. All three objects are made of an identical material. If the proposal by Xiao et al. (2020) generalizes well to all geometries, then the ranking from the most translucent to the least translucent should be the following: a cylinder, low-resolution Lucy, and a high-resolution Lucy. It is difficult to claim the latter definitively. In contrast, we can even speculate that the thin edges of Lucy make it appear more translucent (as discussed elsewhere in this article), its complex surface geometry causes more blur, while other shapes are structurally thicker, flatter, more specular and less blurry. In an earlier work, Xiao et al. (2014) also argue that complex shapes (e.g., the presence of thin and thick parts) generate a greater range of translucency cues and lead to the faster failure of the translucency constancy.

**Figure 14.**
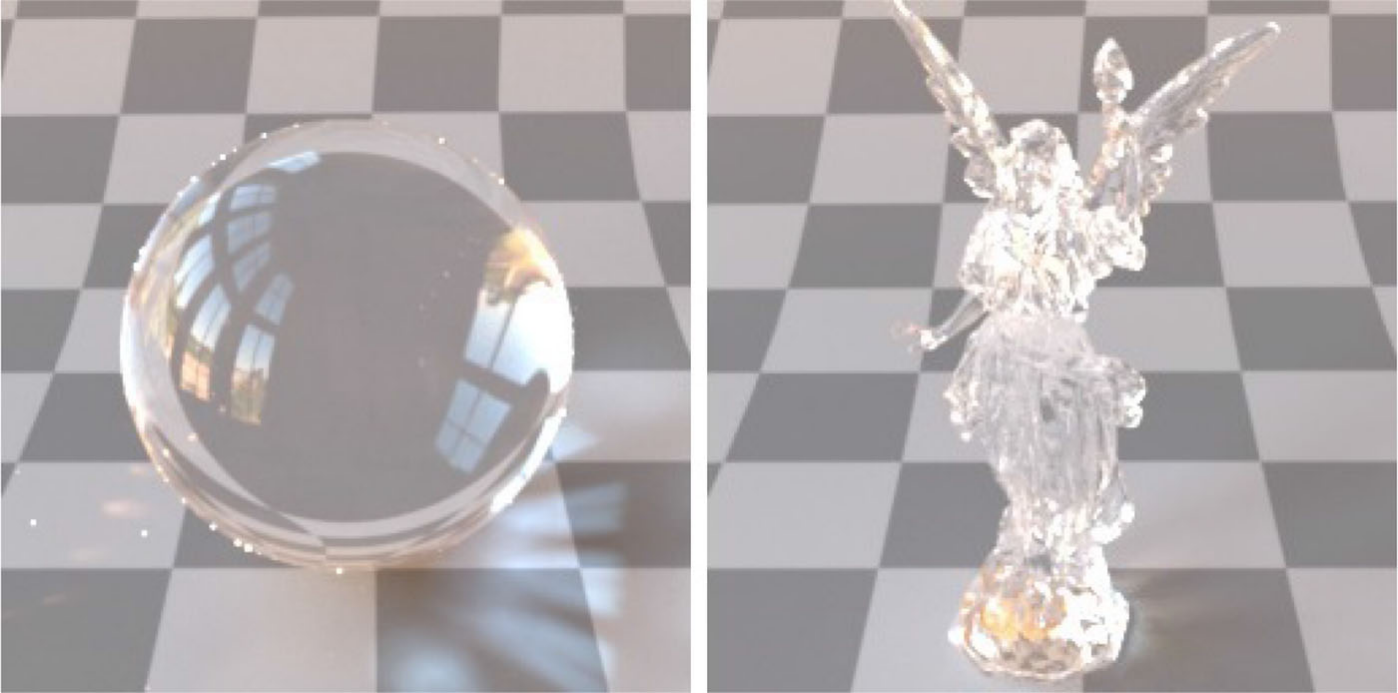
The sphere and the Lucy are made of the identical material. However, while the simple surface geometry and the low local curvature enable observing transmittance image through a sphere, the complex surface geometry and the high local curvature of the Lucy result in more specular reflections, inter-reflections, and caustics. Eventually, although the extinction coefficient of Lucy is 0, its surface geometry makes it impossible to separate surface scattering from subsurface scattering highlights, evoking the feel of translucency rather than transparency. This is especially apparent in tonemapped low dynamic range images, such as those.

Finally, a complex surface geometry might generate more specular highlights, caustics and interreflections — making more difficult to see-through and yielding illusion of subsurface scattering (Gkioulekas et al., 2015). Think of a transparent glass vase that is shiny, due to its complex shape, and looks as if it scattered light under the surface (see more on this in Todd & Norman, 2019). This phenomenon is illustrated in Figure 14. The sphere and the Lucy are made of an identical material. However, the low curvature and the simple shape of the sphere permits seeing-through it (it looks transparent), while the light scatters on the surface of Lucy and hence, it looks more translucent and less see-through.

#### Illumination direction

**Figure 15.**
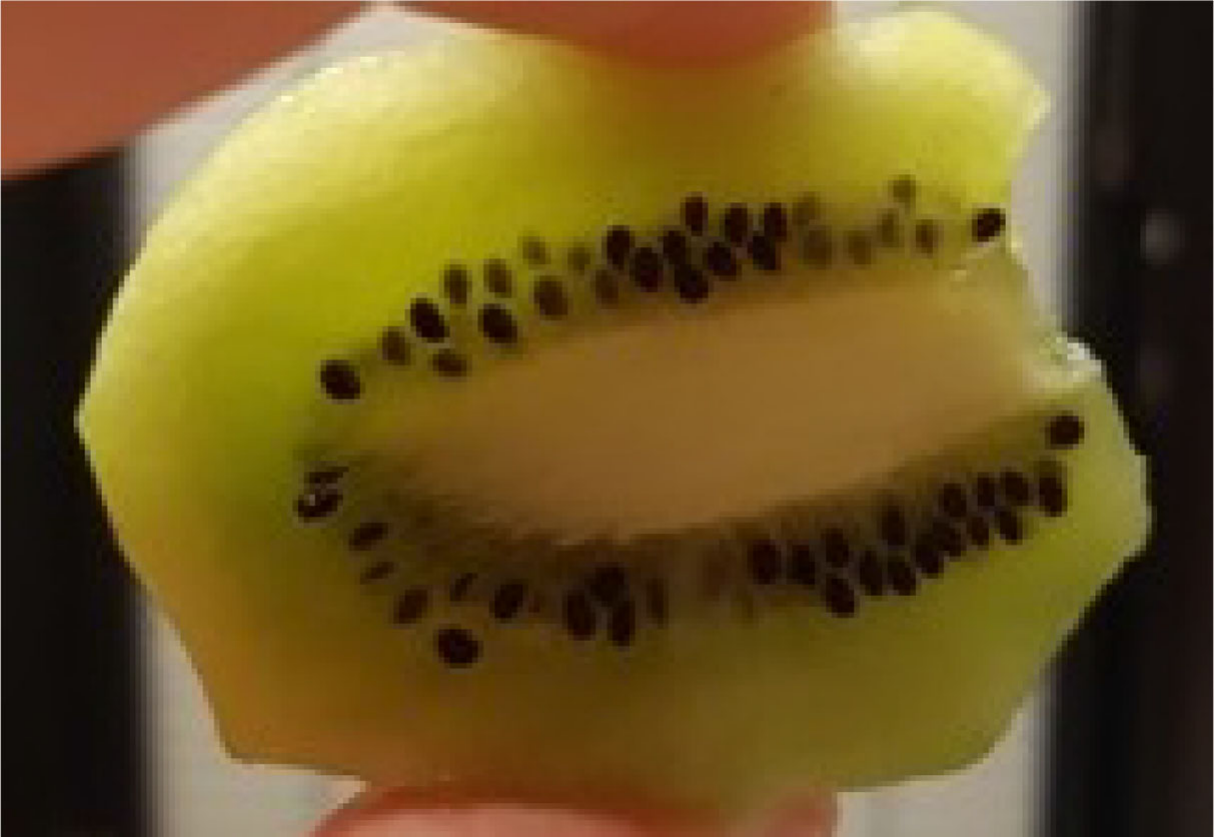
Most fruits look translucent when seen in back-lit illumination geometry. Bright edges and the luminance gradient indicate that the flesh is translucent, while the seeds look solid opaque black.

Illumination direction has one of the most striking effects on the magnitude of perceived translucency. If you have ever taken your food and looked through it toward the sunlight, you should have noticed that it starts glaring (see Figure 15). This effect can be taken advantage of in art and architecture. Also refer to Figure 11, which illustrates the frames from the video (refer to Supplementary Material 1 for the video). Even though the material is identical, the difference in perceived translucency is apparent among the three conditions (compare left, middle, and right images in Figure 11). Koenderink and van Doorn (2001) have argued that translucency is viewpoint-dependent and *“transillumination” of the light* through the material is a strong cue for translucency. Most of the materials look more translucent when the light source and the observer are located in different hemispheres, that is, when a sample is back-lit from the observer's viewpoint. This effect was first illustrated by Fleming and Bülthoff and has been further substantiated experimentally by Xiao et al. (2014), who observed that most materials look more translucent when back-lit and material matching is easier in back-lit conditions than in the front-lit one. Interestingly, Fleming and Bülthoff (2005) report that the information is not diagnostic enough for material discrimination when they are front-lit. This observation is, however, challenged by Xiao et al. (2014), who argue that this can be attributed to using a simplistic torus shape by the authors, whereas in Xiao et al. (2014) experiments with the complex shape of the Stanford Lucy enabled discriminating materials even in the front-lit conditions. Gigilashvili et al. (2018b, 2021b) have observed that humans prefer a back-lit condition for assessing material translucence. They argue that the magnitude of the differences between translucent and opaque materials is larger in back-lit condition — making it a desired geometry for comparing objects. Per contra, in the study of the dental porcelain translucencies (Liu et al., 2010), authors argue that sensitivity toward translucency differences does not differ significantly between front-lit and back-lit illumination conditions. However, the noticeability thresholds are lower for back-lit conditions (with *p* value ≈ 0.06). It has been also observed that textiles that normally look opaque might look translucent when back-lit (Gigilashvili et al., 2019a) — having implications for clothing and curtain manufacturing. Gkioulekas et al. (2013) noted that the illumination direction has the strongest effect on the appearance space where they embed different phase functions. As noted elsewhere in this article (Figure 5), the parameter of the Henyey-Greenstein phase function has the weaker effect under the back-lit illumination condition (compare the top and bottom rows). In contrast, Marlow and Anderson (2021) observed that the intensities produced by subsurface scattering remain relatively stable when the observer and the light source remain in the same hemisphere and the illumination angle changes from orthogonal to low grazing angles.

#### Illumination structure

**Figure 16.**
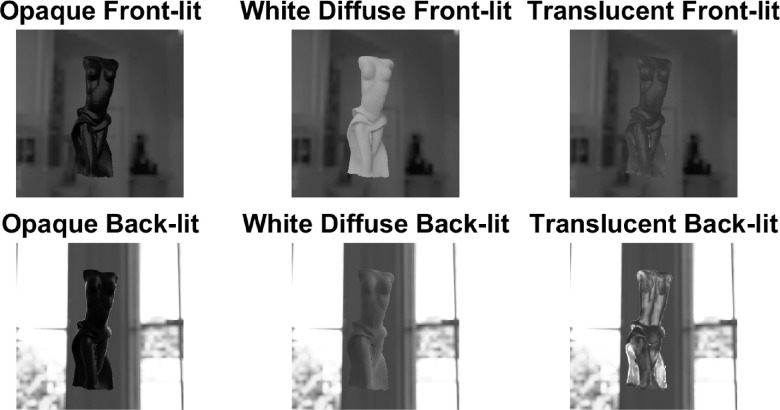
The way the illumination geometry modulates object appearance differs strikingly between translucent, black somewhat specular opaque, and white Lambertian objects.

The impact of illumination structure on the perception of translucency is not well-explored. Although Xiao et al. (2014) argue that it is important to study translucency in the natural complex illumination and not under simplistic point light sources, as in (Fleming & Bülthoff, 2005; Motoyoshi, 2010; Nagai et al., 2013). Intuitively, a collimated beam should penetrate deeper than the diffuse ambient light inside the material and thus, is expected to generate higher magnitude of translucency. This was illustrated by Gigilashvili et al. (2019a). They observed that textile samples were considered opaque under diffuse illumination, although some of them were reclassified as translucent when a high-luminance directional lamp was introduced in the scene. Presence of the shadows, which are thought to be one of the most important cues for assessing translucency (discussed elsewhere in this article), also depend on the illumination structure. For instance, in case of a directional light, the only way shadowed and concave regions can get light is via subsurface scattering, while in case of diffuse and more natural illumination, shadowed regions can receive light also from the ambience, which can impact how translucency or opacity of the material is interpreted (Fleming & Bülthoff, 2005; Nagai et al., 2013; Xiao et al., 2014) (also see white diffuse front-lit and translucent front-lit in Figure 16). Motoyoshi (2010) argues that sometimes it can be difficult to understand whether the blurry appearance is a result of subsurface scattering or diffuse illumination. Marlow et al. (2017) argue that, for distinguishing translucency and opacity, the HVS uses the covariance between surface and shading. If the surface and shading do not covary and the regions which were expected to be shadowed look lighter, a sensation of translucency is generated. They illustrated that, if embedded in a proper light field that generates or eliminates this covariance, it is possible to render an illusory translucency on optically opaque media and the other way round. However, it is important to explore how often this can be encountered in the natural conditions. Fleming et al. (2003) observed that matching accuracy of the surface reflectance properties decreases under nonrealistic illumination, and the random patterns of illumination might not generate glossy appearance at all. Similar phenomena could potentially be true for translucency. However, gloss has been shown to be less dependent on illumination than observed by Fleming et al. (2003), when complex shapes are used and the Fresnel effects are accounted for (Faul, 2019).

#### Caustics

**Figure 17.**
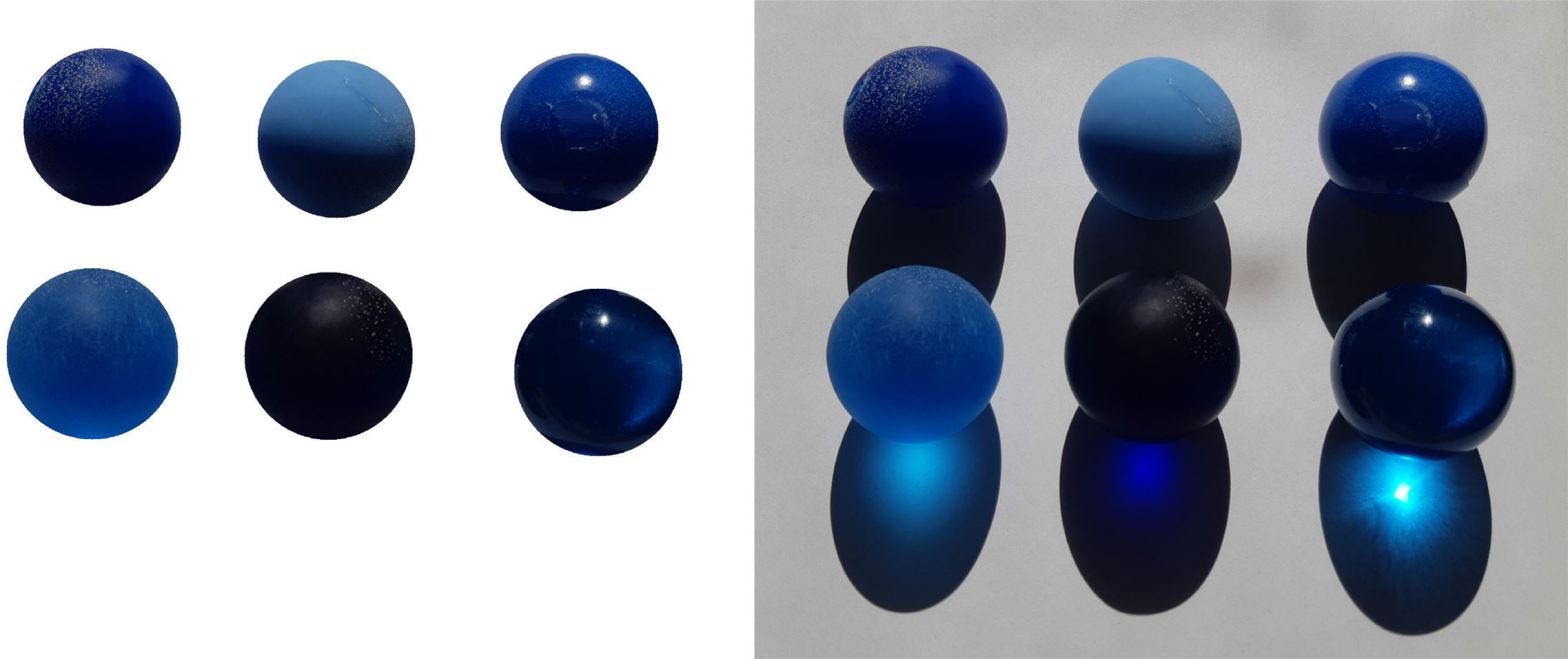
Although the object body might look fully opaque (e.g., the middle object in the bottom row), the external caustics provide rich information about the subsurface light transport properties of a material. The figure is reproduced from Gigilashvili et al. (2019a). The animated version can be seen in Supplementary Material 2.

Although all previous research (e.g. Fleming & Bülthoff, 2005; Marlow et al., 2017; Motoyoshi, 2010) attempted to identify translucency cues on the object body proper, Gigilashvili et al. (2018b, 2021b) noticed that, for assessing translucency, human observers put an importance on the cues elsewhere in the scene — primarily, the caustic patterns that are cast by an object onto another surface. The shadows cast by translucent and opaque objects differ (compare the top and the bottom rows in Figure 17). In some particular scenarios, caustics might be the only indicator of translucency, while from the object body alone, it might be impossible to infer that (compare the middle object in the bottom row between the left and right images of the Figure 17). Gigilashvili et al. (2020a) have shown experimentally that placing an object on a black surface and eliminating the caustic pattern cast onto that decreases perceived magnitude of translucency. Whether this is solely attributed to the absence of caustics, or the impact of the ambient surface on the overall luminance of the object also contributes to that effect should be the topic of the future research.

#### High-level cognitive understanding

It has been shown that appearance perception is not a one-way pipeline, but rather a loop where the low-level vision is not simply an input for mid- and high-level vision, but also gets impacted by them (Anderson, 2011; Bartleson, 1960). Chadwick et al. (2018, 2019) made two interesting observations: although translucency perception is anatomically independent from color perception, observers with normal vision are still better at judging translucency in color images than in their grayscale counterparts, which could potentially be attributed to the easier identification of the familiar materials; second, people estimate absorption and scattering properties better in the stimuli existing in reality than in synthetic, virtual materials — proposedly attributed to a better training and experience with interacting with the real materials. Prior experience might be a considerable factor when assessing translucency. For instance, Liu et al. (2010) studied translucency perception of dental porcelains and found that the experts with “more than 10 years of shade-matching experience” discriminate levels of translucency better than novice students. In contrast, Motoyoshi (2010) reported that there has been no difference between the observers who had seen and who had not seen the experimental stimuli before the experiment. Nagai et al. (2013) observed cross-individual differences in translucency cues. The authors used psychophysical reverse-correlation methods and found that different people looked at different regions of the objects to assess translucency, however, the exact reason remains unknown. The vast majority of the observers looked at the face of the Stanford Buddha shape used in the experiment, even though it might not have been the most informative region in terms of image statistics. According to the authors, this could be attributed to the fact that the human face catches attention easily (Hershler & Hochstein, 2005).

The high-level cognitive information seemingly plays a role in the perception of painterly translucency. It has been shown that depiction and perception of translucency is related with the perceived realism and “convincingness” of the artworks (Di Cicco et al., 2020b; Wijntjes et al., 2020). Wijntjes et al. (2020) hypothesize that high-level cognitive factors might be contributing to perception of translucency in the sea paintings, such as an priori expectation that the water in the Caribbean scenes should be more transparent and translucent, than in the depictions of the nontropical regions. Gigilashvili et al. (2018b, 2021b) noticed that observers try to identify materials when assessing their translucency and glossiness. The convincingness of translucency is enhanced with glossiness, proposedly due to the memory of familiar objects (Fleming & Bülthoff, 2005). Material perception has been shown to be a multimodal process relying on multisensory information (Spence, 2020). If material identification contributes to translucency perception, this opens up a new question, whether the senses other than vision play a role in the perception of translucency, either directly or indirectly. Marlow et al. (2017) have demonstrated that translucency perception to some extent implies understanding and estimating surface geometry. Additionally, when observing an object with varying thickness, we are able to perceive the object as made of a single, homogeneous material and not a composite of different materials, even though the luminance statistics and other translucency cues might differ considerably among these regions. All these observations indicate that translucency might not depend solely on the low-level vision cues, but that high-level cognitive factors might be contributing to that as well. The fact that people, for instance, understand and use caustics (Figure 17) for inferring translucency, already involves a high level cognition of the scene. How much perceived magnitude of translucency is impacted by the high-level vision should be answered by future research.

#### Motion and scene dynamics

A fundamental problem in translucency perception is separating reflected and transmitted energy in the proximal stimulus on the retina. In this process, the HVS can obviously benefit from understanding the distal stimulus — the scene and the ambience. Motion has been demonstrated to be important for gloss perception and gloss constancy (Doerschner et al., 2011; Hartung & Kersten, 2002; Wendt et al., 2010), especially for separating specular highlights and surface texture. Additionally, motion can help with understanding the object shape and geometry. In contrast, the energy emerging from an object after subsurface scattering depends on the spatial location, making translucency viewpoint dependent, as noted by Koenderink and van Doorn (2001). Therefore, observing a translucent object from different viewing geometries should intuitively provide additional information about the bidirectional surface scattering reflectance distribution function. Van Assen et al. (2018) have demonstrated that motion is important for perceiving viscosity and elasticity of translucent liquids and spreadable materials. Fleming (2014) hypothesizes that the HVS learns and predicts how appearance of a given material varies across different conditions, inherently implying motion in the learning process. Gigilashvili et al. (2018b, 2021b) analyzed human behavior when they were asked to assess translucency. They observed that humans frequently use motion-related cues; they move the fingers behind the object, move the object over a textured surface, move it relative to the light source and compare the object's appearance between front-lit and back-lit conditions. In short, it is natural for humans to change the background, observe how much it has impacted the appearance of an object and infer light transmission properties from it. This is qualitatively related to the phenomenon of change blindness in image quality, with the change being more apparent when the subsequent frames are toggled back and forth, rather than being observed on independent occasions (Le Moan & Pedersen, 2017). Fleming (2014) hypothesizes that the brain might be building a statistical generative model of appearance that first learns and then predicts how appearance of a given material varies across different natural illumination conditions. If this hypothesis is true, interaction and dynamics would be an inherent part of the learning process from the infancy age. However, we do not know what part of it is learned and what is inherited.

The impact of motion is demonstrated in the video available in Supplementary Material 1. The video shows that motion relative to the illumination has a considerable impact on the luminance distribution on the object body and makes perception of translucency more convincing. Xiao et al. (2014) argue that motion might enhance material and translucency constancy. Intriguingly, although translucency constancy fails due to the illumination direction change, the continuous motion in the video (Supplementary Material 1) enables material constancy; we understand that it is the same material and its appearance changes owing to the illumination, not owing to the change in the optical properties of the material.

To the best of our knowledge, Tamura et al. (2018) have been the only ones to empirically study the role of scene dynamics on transmission perception. They found that the relative motion of the image superimposed on the object is significantly important for distinguishing reflective opaque mirrors from translucent glass materials. This once again highlights that still images might not be able to reveal the full range of the cues used by the HVS.

### The role of other appearance attributes

#### Color

When talking about color, it is crucial not to mix up the chromatic and achromatic components. Lightness or brightness are directly correlated with the absorption and scattering, which make materials look darker or brighter, respectively (Chadwick et al., 2018; Cunningham et al., 2007; Koenderink & van Doorn, 2001; Urban et al., 2019) (see Figure 2). As many translucent materials we interact with on a daily basis, such as milk, cream, cheese, and snow, have a whitish bright diffuse-looking appearance, Gigilashvili et al. (2021b) have observed that many observers associate lightness with milkiness and translucency. However, lightness information is certainly subject to spatial and geometric constraints (Marlow et al., 2017). For instance, brighter edges (Di Cicco et al., 2020b; Fleming & Bülthoff, 2005; Gkioulekas et al., 2015; Wijntjes et al., 2020) and shadowed areas (Fleming & Bülthoff, 2005; Marlow et al., 2017; Motoyoshi, 2010) are direct indicators of translucency. For completeness’ sake, we should mention that Sawayama et al. (2019) found that a mean color difference between the images is, indeed, not informative enough to discriminate translucency.

**Figure 18.**
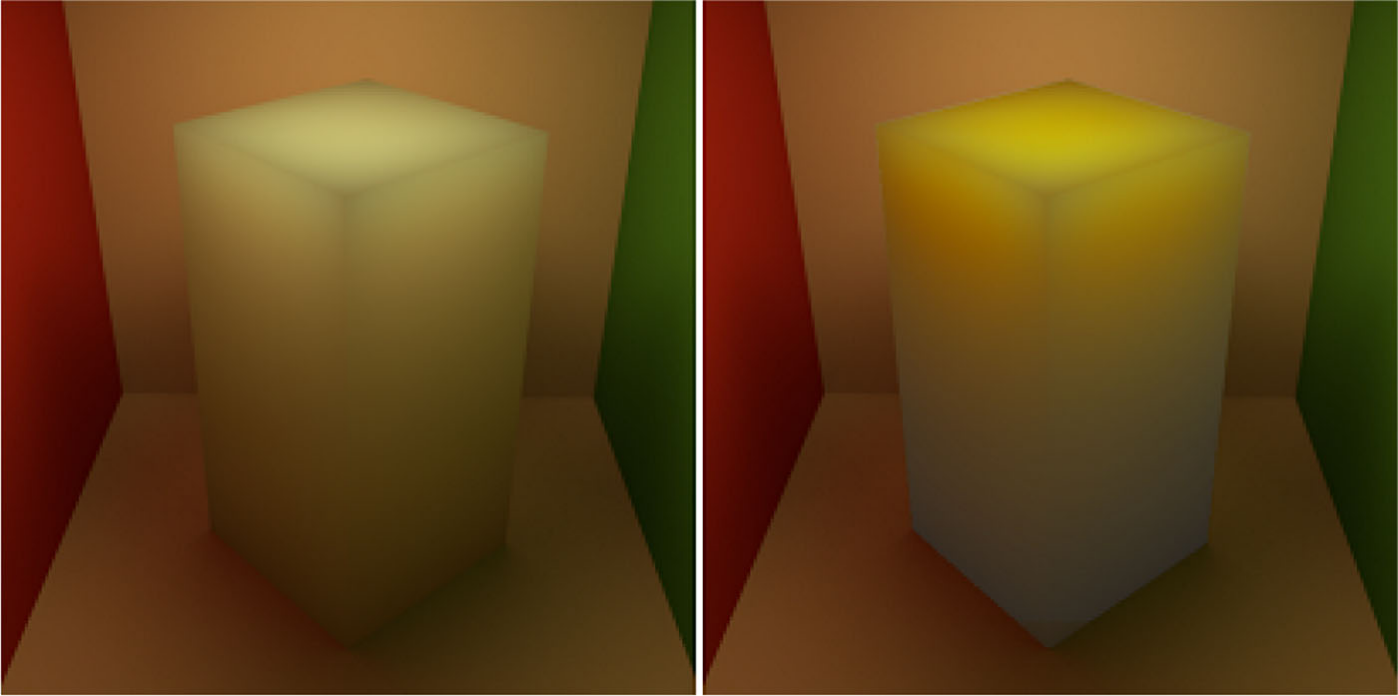
The mean saturation is equal in both images. However, the figure in the left image has a negative correlation between saturation and value (of HSV), whereas the right one has a positive correlation. This, as observed by Fleming and Bülthoff (2005), makes their translucence glow icier and warmer, respectively.

In contrast, little is known about how chromaticity contributes to translucency. Chadwick et al. (2019) have worked with an observer who has a color deficiency of a cortical origin. They demonstrated that color and translucency processing happens in the different parts of the brain and, thus, are anatomically independent. However, they also observed (Chadwick et al., 2018, 2019) that the color normal observers perform better on color images rather than on grayscale ones, potentially explained by higher level cognitive processing related to the material identification and realism. Di Cicco et al. (2020c) have recently shown that perceived translucency of painted citrus fruits is significantly correlated with their color saturation. Fleming and Bülthoff (2005) have illustrated that saturation might enhance the effect of translucency. Namely, if the saturation and lightness intensity are correlated positively, translucency looks like a warm glow, while it looks icy translucent in case of the negative correlation. This phenomenon is illustrated in Figure 18. Moreover, the perception of wetness, which is optically related to translucency, has been also shown to be related with saturation (Sawayama et al., 2017). However, it is noteworthy that saturation alone cannot evoke perception of translucency. Besides, absorption and scattering coefficients of the most natural materials are wavelength dependent, a phenomenon used extensively in 3D printing (Brunton et al., 2018, 2020) and art (Gigilashvili et al., 2021b; Thomas et al., 2018). Therefore, the amount of the light emerging after the subsurface light transport will depend on the spectral power distribution of the illuminant. For instance, if the material that fully absorbs red wavelengths is illuminated with a red light, it might look opaque, not translucent. Although the effect might be negligible and rare under natural illumination, potential aesthetic effects generated with spectral translucence deserve future search and exploration.

#### Gloss

It has been shown that translucency impacts apparent gloss (Gigilashvili et al., 2019b, 2021a). However, the correlation the other way round is not clear and straightforward. Moreover, Schmid et al. (2020) argue that the neural aspects of gloss perception should be addressed in the context of material identification, highlighting the resemblance of the visual features between material recognition and glossiness perception. Schlüter and Faul (2019) argue that specular reflections have an important implication for the perception of transparency. There are several indications in the literature that glossiness might be increasing perceived magnitude of translucency. This phenomenon has been observed by Motoyoshi (2010) (although no effect was observed by Nagai et al., 2013). Furthermore, Yu et al. (2019) have proposed a highlight-generation method for rendering translucent appearance. Although the primary intention was to enhance the perception of the fine details, interestingly, the perceived magnitude of translucency was also enhanced. Translucency and glossiness have been observed to be positively correlated in paintings (Di Cicco et al., 2020b; Di Cicco et al., 2020c; Wijntjes et al., 2020). Fleming & Bülthoff (2005) have observed that glossiness enhances the realism of translucent appearance, potentially attributing to the fact that many translucent materials are also glossy (e.g., glass, marble, liquids), and we “expect” translucent objects to be glossy. However, translucency and gloss cannot alone explain each other, because many glossy materials, such as metals, are not translucent (Fleming et al., 2013; Tanaka & Horiuchi, 2015) and many translucent materials, such as smoke, cotton, and textiles, are not glossy (Koenderink & van Doorn, 2001). For instance, in Figure 8 the top row of the objects, which lack specular reflections owing to a negligible change in the refractive index look smokey, or spongy, but still vividly translucent. The bottom row possesses the identical subsurface scattering properties, but adds specular reflections owing to a large change in the refractive index. We can argue that the bottom row looks more realistic and more likely to be encountered in real life, but any estimation of the perceived magnitude of translucency, unless the difference between the refractive indices is large (see Figure 7), would be purely speculative. In some cases, the correlation between gloss and translucency can be straightforwardly negative, because the surface roughness, which decreases the magnitude of glossiness (Pellacini et al., 2000; Thomas et al., 2017) itself evokes the perception of translucency (refer to Figure 12). Moreover, the increase in the refractive index generates a stronger Fresnel reflection, that is, stronger glossiness and less transmittance (Koenderink & van Doorn, 2001), as illustrated in Figure 7. Finally, glossiness and specular highlights can facilitate understanding the shape (Fleming & Bülthoff, 2005; Fleming et al., 2004; Marlow & Anderson, 2021; Norman et al., 2004; Todd & Norman, 2003; Xiao et al., 2014). As the shape comprehension is proposedly related to translucency perception (Marlow et al., 2017; Marlow & Anderson, 2021), gloss can play a supplementary role in this manner too. Marlow and Anderson (2021) have recently identified covariance between the intensity gradient produced by the subsurface scattering and the shape of the specular reflections, both helping the recovery of the 3D shape and material properties. Moreover, they have experimentally shown that a light-permeable surface covered with convex and concave regions is perceived more translucent when physically accurate specular reflections are superimposed. However, the effect is weakened or lost if the reflections are rotated and thus, incongruent with the subsurface scattering gradient.

## Cues for translucency perception

These intrinsic and extrinsic factors are impacting the proximal stimulus in a way that the HVS can deduce subsurface scattering and light transmission in the images. Although the scene dynamics and the temporal aspects enhance translucency detection, it is possible to perceive translucency from still images, which makes the researchers conclude that there should be some diagnostic features and statistics in the 2D images, which separate translucent media from the opaque ones. For example, it has been proposed that the skewness of the luminance histogram might be correlated with perceived gloss (Motoyoshi et al., 2007) (but see Anderson & Kim, 2009; Kim et al., 2011). There have been attempts to identify similar measures diagnostic for translucency and to propose at least partial models of translucency perception. Singh and Anderson (2002a) argued that, in see-through scattering media, both apparent contrast and apparent blur of the background contribute to the perception of translucency. However, the cues on the objects that did not permit seeing a background through them remained largely unexplored. Although no full model of translucency perception exists, and none is close being as complete as the Metelli-type models of transparency, several interesting observations have been made in the past 15 years, which reveals some interesting characteristics of the translucency perception mechanisms. We overview these partial models and also provide some illustrations based on the *bust* renderings from the *Plastique Artwork Collection* (Thomas et al., 2018), which is rendered in the Mitsuba-embedded natural illumination (Jakob, 2010). Using this shape for the demonstrations has two practical implications: first, it has a varying degree of structural thickness, sharp edges and fine details, providing a broad range of translucency cues; and second, a behavioral study has been conducted on the physical replica of this shape (Gigilashvili et al., 2018b, 2021b), which permits comparison of the real and synthetic stimuli in the future. We believe that this shape could become a standard for translucency perception research in parallel with Stanford Lucy (Stanford University Computer Graphics Laboratory, 1994). In the demonstrations below, we will mostly rely on a comparative analysis of six intensity images of the *Plastique bust* shape: a highly translucent material (referred to as *“translucent”*), highly absorbing somewhat specular black opaque material (*“black opaque”*) and a Lambertian-looking white diffuse opaque material (*“white diffuse”*) in back-lit and front-lit illumination conditions. These images are shown in Figure 16.

### Fleming and Bülthoff

Fleming and Bülthoff (2005) were the first ones who tried to model the perception on the non-see-through scattering media. They have noticed that the intensity gradients differ between opaque and translucent objects, where the largest difference is noticeable near the edges. Bright and blurry edges are usually characteristic to translucent objects. Simplistic image manipulations by adding those features to a Lambertian surface using a high-pass filter enabled the authors to generate some degree of translucency, although not very realistic looking (see Figure 14 in Fleming & Bülthoff, 2005).

**Figure 19.**
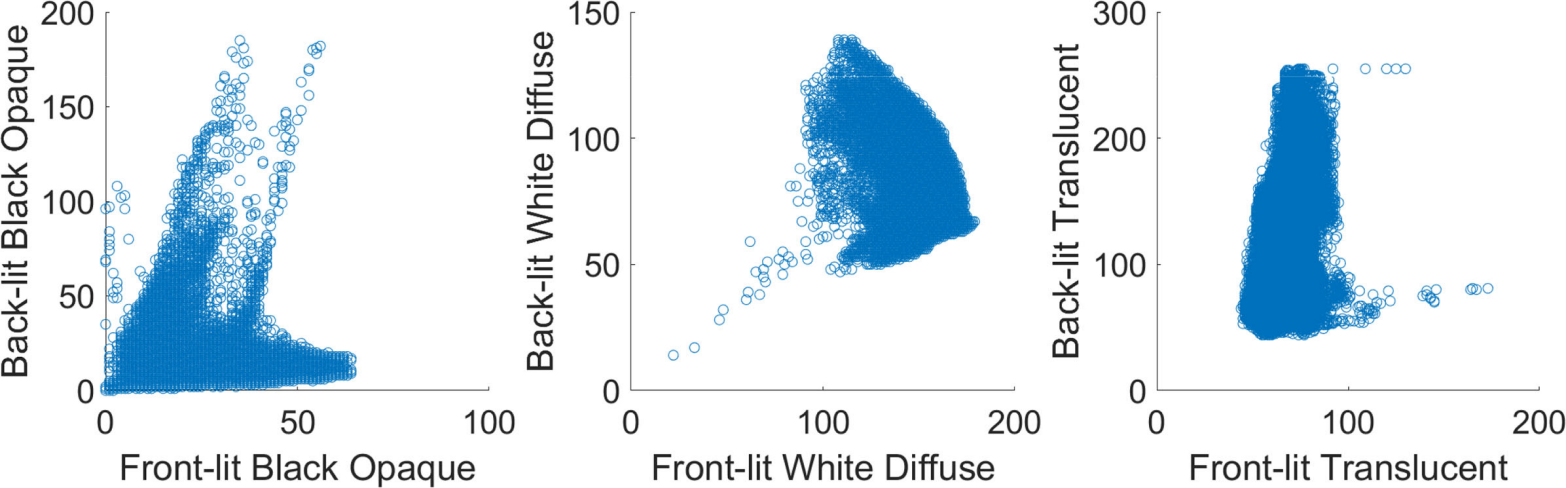
The correlation of the intensities between the identical pixels of the same object under two different lighting conditions. The plots show that, although far from being linear, the dependence is not random. Most pixels of the black opaque object are simply darkened as they fall in the shadow when the light is moved on the back side. Some intensities, mostly on the edges, increase, because the backlight is not incident fully perpendicularly, and some of the light comes from the side angles as well. For the diffuse white object, the relationship is usually negative, except for some pixels on the edge, that brighten under back-light and are thus, positively correlated. For the translucent object, the slope is steep and the values simply go up when the object is placed under the back-light. We identified that the behavior of the pixel intensity is strongly dependant on its spatial location. However, the overall trend differs between the three objects and the change of pixel-wise intensities between the illumination geometries might to some extent indicate to the subsurface light transport.

**Figure 20.**
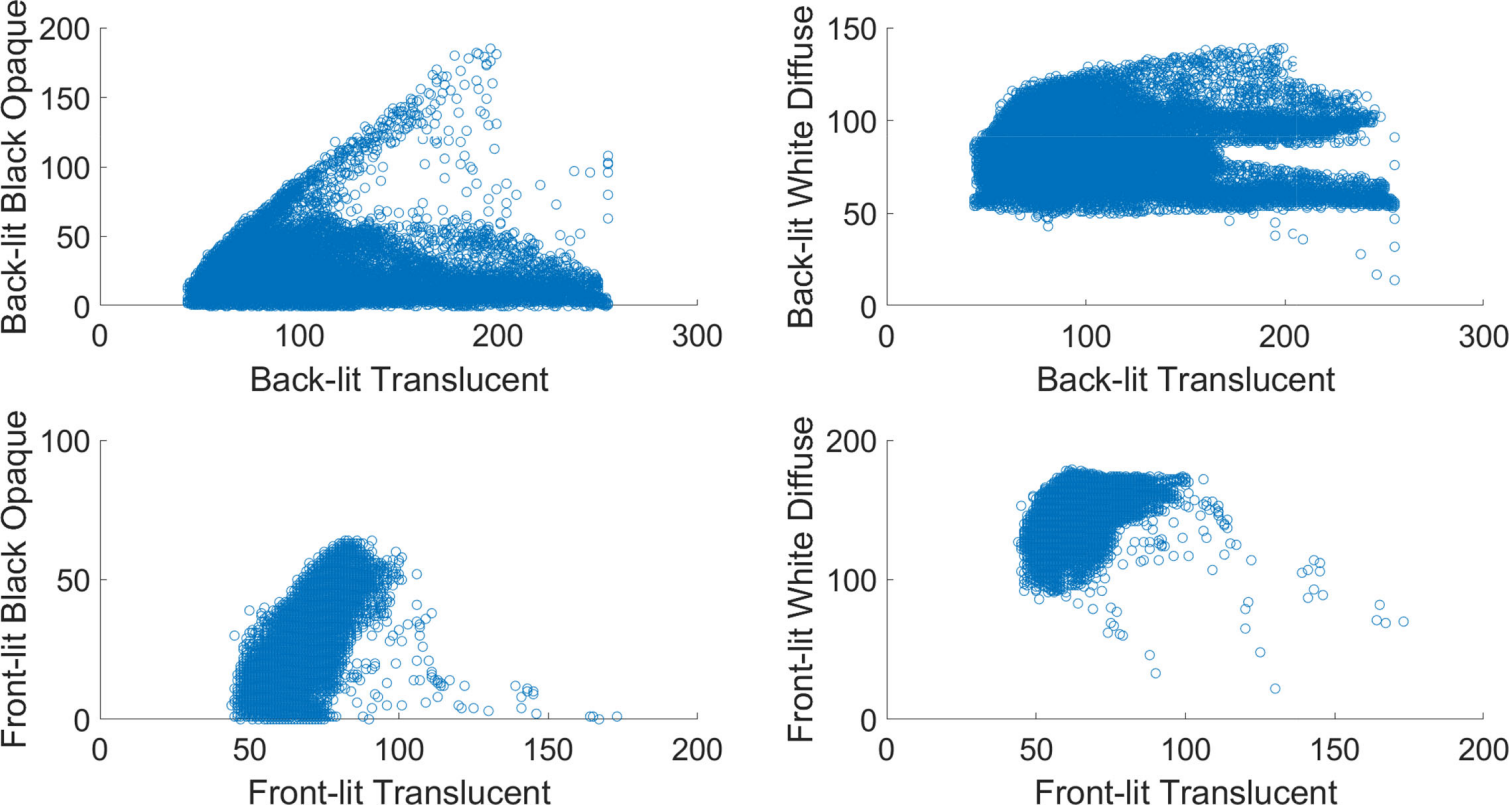
The figure illustrates pixel-wise correlation of the intensities under a given illumination geometry between a translucent object and other two non-transmissive materials. Although the correlation looks mostly random under the back-lit condition, it becomes more visible when the objects are front-lit. The pixels become dimmer on the black opaque object, because its nonspecular areas simply absorb light, whereas nonspecular areas of a translucent object either back-scatter some of it, or transmit from a background toward the camera. The opposite is true for the white diffuse material, because more light gets scattered toward the camera by a white opaque object and no energy is lost due to the subsurface scattering away from the camera (the similar phenomenon was observed by Nagai et al., 2013).

They also observed that the contrast between the specular and nonspecular regions is smaller for translucent objects and on the example of a simple torus image, they demonstrated that the histogram of an opaque object is more skewed (e.g., compare the first two columns in Figure 27). They observed that pixelwise-correlation between translucent and opaque images is far from linear and it alone cannot be a predictor for translucency. We have plotted how the intensity values change for each pixel of an identical material across two different conditions (see Figure 19) and between different materials under the same illumination (Figure 20). Similar to Fleming and Bülthoff, we also observe that the correlation is not random, but highly nonlinear (see Figures 19 and 20). For instance, when the illumination direction changes, the slope is steeper for a translucent object, while an opaque object intensities remain less impacted. The effect illumination geometry has on a pixel's intensity of a given material strongly depends on the spatial location of this pixel. We also noticed that in a back-lit illumination condition, the correlation between translucent object intensities and the opaque ones is mostly random, because of the high magnitude transmission component. In contrast, in the front-lit condition, the nonspecular spatial locations of a translucent material are lighter than their black opaque material counterparts, but darker than white diffuse ones (cf. captions of Figures 19 and 20).

**Figure 21.**
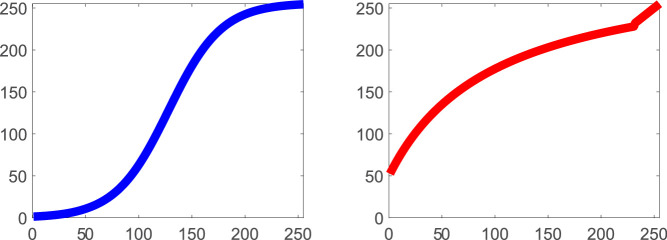
The sigmoid function (left) stretches low and high intensity values towards the extremes, which increases the overall luminance contrast. The “N-shaped” curve (right) scales up lower intensities, and keeps the highlights intact, decreasing the contrast between specular and shadowed areas. Fleming and Bülthoff (2005) observed that under fixed illumination conditions, similar functions can be used to enhance opacity and translucency, respectively.

**Figure 22.**
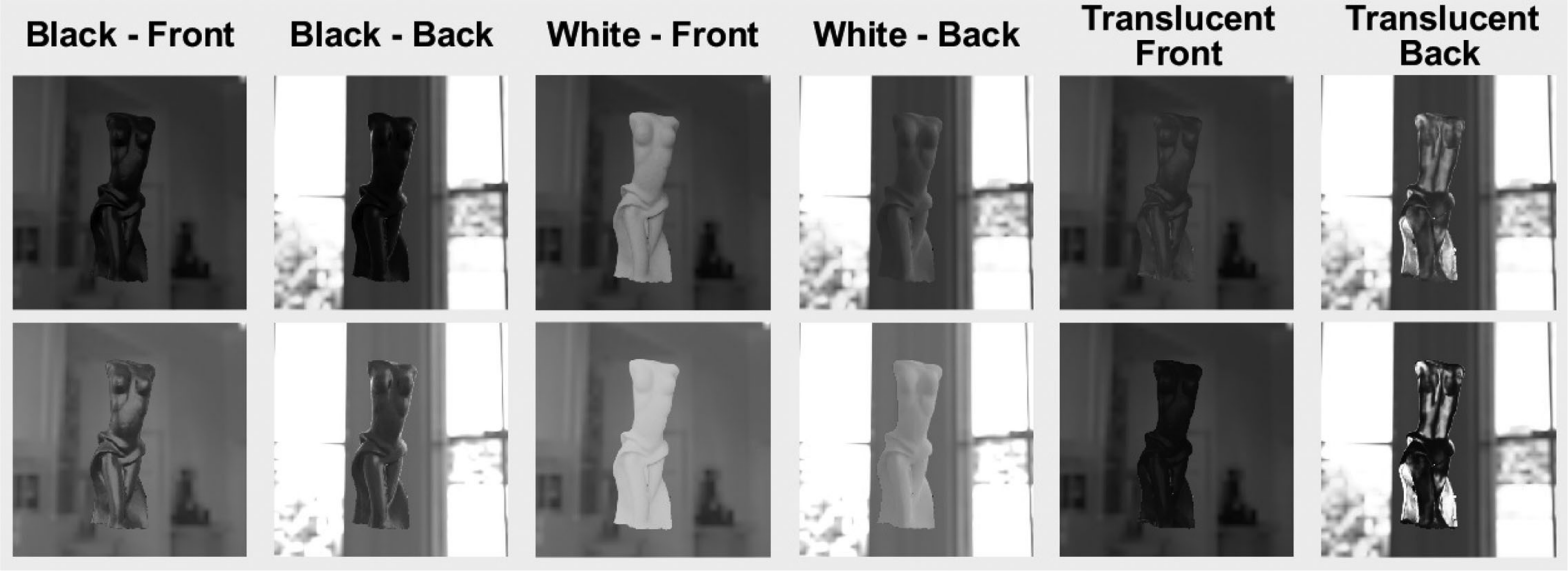
The nonlinear functions (shown in Figure 21) applied to the intensities. We tried to make opaque objects more translucent with an “N-shaped” function, and a translucent object more opaque with a sigmoid function. The top row illustrates the original image intensities and the bottom row shows the results after application of the nonlinearity. The front-lit black opaque object has become slightly more translucent looking because the contrast between the specular and nonspecular regions has decreased. However, it can also be interpreted as an opaque object of a simply lighter shade. The back-lit black object does not look transmissive, but rather white diffuse material (compare with *White - Back* in the top row), because scaling up darker shades makes it more reflective but it is unable to generate the transmission gradient similar to that of a translucent object (compare with rightmost images in both rows). The translucent look of a front lit white diffuse material has been considerably enhanced, because making shadowed areas lighter creates the feel that *“photons could not get there without subsurface scattering.”* Interestingly, under backlight, although it looks less opaque, it does not process gradient characteristic for transmission or subsurface scattering either, making it look somewhat unrealistic. On the other hand, as the immediate background of the object is a wall, not the light source proper, its dim color can also be interpreted as a thin transparent filter. Finally, the front-lit translucent object became more opaque by eliminating the lighter shades in the nonspecular areas and no cue has been left that could hint the HVS to the subsurface light transport. However, the approach failed in back-lit illumination geometry. Although the increased contrast between lights and darks make it look more solid, the highlights that are usually thought to be specular reflections, are transmission components in this case and scaling them up strengthens the perception of transmission. This was observed by Motoyoshi (2010), who noticed that in transparent materials and thin parts, the contrast is reversed or random. This illustrates that simple context-blind nonlinear scaling does not control translucency-opacity appearance. In the rightmost image in the bottom row, we do not perceive highlights as specular reflections. We somehow understand that this is the result of light transmission. Therefore, the higher level cognitive mechanisms of the scene and shape understanding seem to be involved in the translucency perception process.

**Figure 23.**
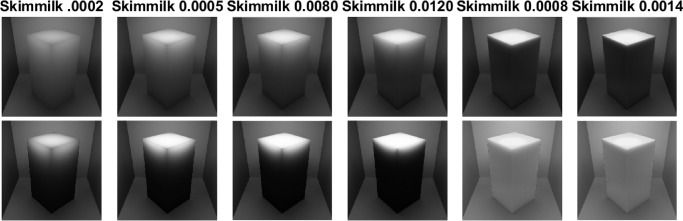
The nonlinearities identical to that in Figure 22 applied to simpler shaped objects. The top row illustrates the original intensities, and the bottom row is the nonlinear filtered result. The contrast enhancement darkened the shades in the bottom of the box, where light penetration is little. However, it made the near-edge areas brighter, which, in the first four columns, still produces somewhat unrealistic feel of translucency. The translucent look feels more and more unrealistic with the increase in the optical density (e.g., compare the first and the fourth images in the bottom row). On the other hand, an interesting result was produced by scaling up the lower intensities in nearly opaque objects (two columns on the right). Because the side of the box that faces away from the illumination looks lighter, it overall evokes a feel of highly scattering bright material. This effect is stronger in column 5. Interestingly, keeping the original highlights on the side that directly faces the incident illumination made them look more like specular reflections.

Nevertheless, Fleming and Bülthoff (2005) were able to enhance translucency by applying a carefully selected “N-shaped” filter and to enhance opacity by applying a sigmoid filter to the intensity values. However, they note that this approach can only work when lighting is fixed and spatial correspondence between the pixels is unchanged. The authors illustrated isophotes — the contours of equal lightness and concluded that neither luminance distribution histogram, nor the spatial isophotes, can predict translucency alone, but it is rather more likely that the HVS relies on a combination of the luminance and spatial information. We came up with the qualitatively similar nonlinear filters (shown in Figure 21) and tried to use them for making opaque objects translucent and translucent objects opaque (refer to Figures 22 and 23). We noticed that while the approach might work to some extent (especially, in the front-lit condition), it fails in the thin parts, especially in the back-lit condition. Because Fleming and Bülthoff used a simple torus shape in their study, we tried the approach on a simple shape as well, such as a parallelepiped cube, which also produced considerable artifacts near the edges (refer to Figure 23). In the back-lit condition, the thin parts are bright, which is even further enhanced with a sigmoid filter. However, interestingly, unlike the front-lit condition, we do not see highlights in the thin areas as specular reflections. We somehow understand that they are a result of the subsurface light transport, which makes us believe that in addition to the low-level image cues, the higher order cognitive processes of the scene and geometry understanding also play a role in the translucency perception pipeline (refer to the captions of Figures 22 and 23).

**Figure 24.**
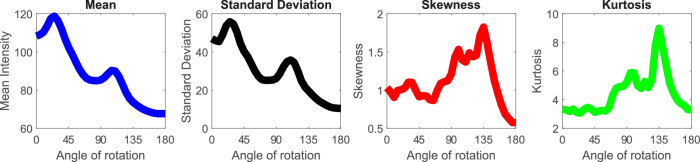
The back-lit bust figure is rotated with 180° all the way to the front-lit condition. The plots show how the first four moments of the intensity histogram change as a function of the angle of rotation. In the original frame, the wall is the immediate background of the object. Once it is “flies” over the window, its mean intensity and standard deviation go up. They generally go down with the angle of rotation, but one local maxima is noticeable around 120°, because there is another window in the scene, which once again “lights up” the object. The skewness and kurtosis have an apparent peak around 135°. This is the result of a highlight produced by internal caustics. When the object is lit from the left, most of its body looks relatively darker, but a bright strip of the caustic pattern is created on its right side, as a result of photon accumulation. This is visible in the middle frame of Figure 11. This highlight generates the unexpected skew in the histogram. For the front-lit condition, the skewness and kurtosis drop dramatically, as the overall object looks blurry and more homogeneous. It is worth noting that these statistics are non-monotonous, and too dependent on the object shape and scene composition that makes their robustness as translucency cues questionable.

Fleming and Bülthoff also demonstrated experimentally that back-lit objects look more translucent than the front-lit ones and tried to identify which image cues explain this psychophysical variation best. They found that neither pointwise correlation nor the first four moments of a luminace histogram (mean, variance, skewness and kurtosis) are predictors of translucency. In order to test this observation, we have rotated a bust figure with 180° from back- to front-lit condition and visualized the summary statistics of a luminance histogram as a function of the rotation angle (refer to Figure 24). Similar to Fleming and Bülthoff, we also noticed that they are nonmonotonous, and although some trends can be identified, they are prone to bias owing to the object shape and the distal stimuli in the scene composition, which makes them unlikely and unrobust cues for translucency perception (refer to the caption of Figure 24).

**Figure 25.**
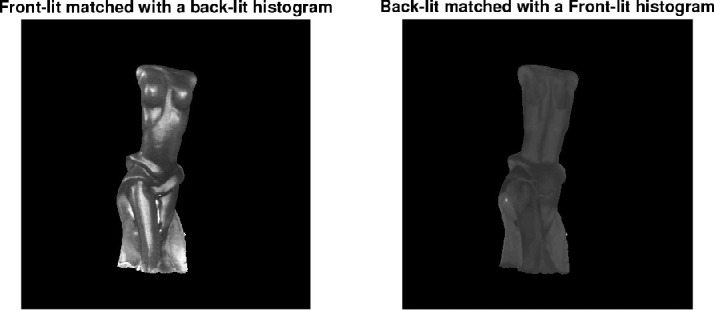
If we match the histogram of a front-lit translucent object with its back-lit counterpart, it starts looking opaque. This can be attributed to the highlights that were the result of trasnmission under the backlight but look more like specular reflections under the front-side illumination. However, some artifacts are still visible near the edges, which look unnaturally specular. On the other hand, owing to the strong transmission gradient, we have not been able to produce an opaque look with a back-lit object, but blur in the highlight areas produces less transmissive but highly scattering look.

Because neither histogram nor spatial information alone are enough for predicting translucency, we tried whether simple histogram matching between front-lit and back-lit conditions of the same translucent material could affect their appearance. Histogram matching affects the magnitude of intensities, but is also to some extent “spatially aware.” The resulting images are shown in Figure 25. Although some artifacts were produced (e.g., near the edges), matching the front-lit object with its back-lit counterpart enhanced its opacity, because the high transmission pixels in a back-lit object, can be interpreted as specular reflections in the front-lit scenario. When the back-lit image was matched with the front-lit histogram, it started looking less transmissive, but still highly translucent.

**Figure 26.**
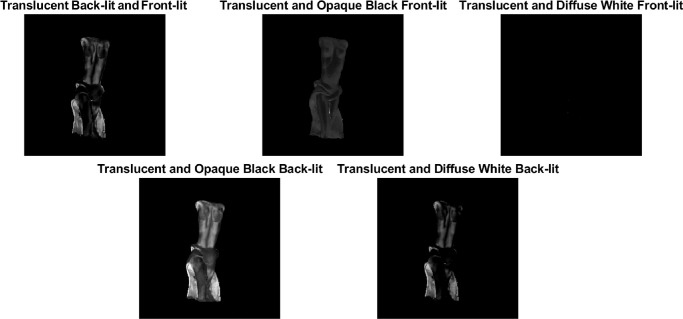
The intensity difference highlights the spatial regions where more energy emerges from a translucent object than from its opaque counterparts (note that this is not an absolute difference between the two images). The first image additionally shows the difference between back-lit and front-lit conditions. Under back-lit conditions, the intensity is higher in virtually every region when compared with a black opaque object, in thin and geometrically flatter areas when compared with diffuse white and itself under front-light. The front lit translucent object has higher intensity in nonspecular regions only when compared with a black absorbing material, while none of its regions have higher intensity than a white front-lit Lambertian object.

Furthermore, Fleming and Bülthoff (2005) argue that the shadowed areas manifest the largest difference between the front-lit and back-lit scenarios and if subsurface scattering is the only way a photon could get to a bright region in the image (otherwise, it would have been in a shadow), that can be used as a cue to translucency. Although this might be commonplace for directional lighting conditions, which renders sharp shadows on opaque objects, Xiao et al. (2014) argue that in diffuse and more natural light fields, the shadowed areas and surface concavities also receive light from the ways other than subsurface scattering. For instance, refer to Figure 26, which highlights the regions where a translucent object has a higher intensity than its opaque counterparts under the same illumination. A front-lit translucent object has larger intensities than its black opaque counterpart in nearly all regions apart from the specular reflections. However, under the same condition, that is not true for a white Lambertian-looking opaque object, which owes its larger intensities in shadowed areas to its highly scattering surface, direct front–side illumination and interreflections. Therefore, the lightness of the shadowed areas alone can not be an indicator of translucency either. In contrast, it is worth noting that the intensity difference between back-lit and front-lit versions of the same translucent material (also shown in Figure 26) could be one of the reasons why back-lit objects look more translucent than their front-lit versions. The authors conclude that the HVS relies on this kind of image cues rather than inverse optics. Indeed, “there is simply not enough information available to invert the actual physics of image formation,” as well-noted by Anderson (2011), but whether the HVS is completely unaware of the laws of physics remains yet to be explored.

### Motoyoshi

**Figure 27.**
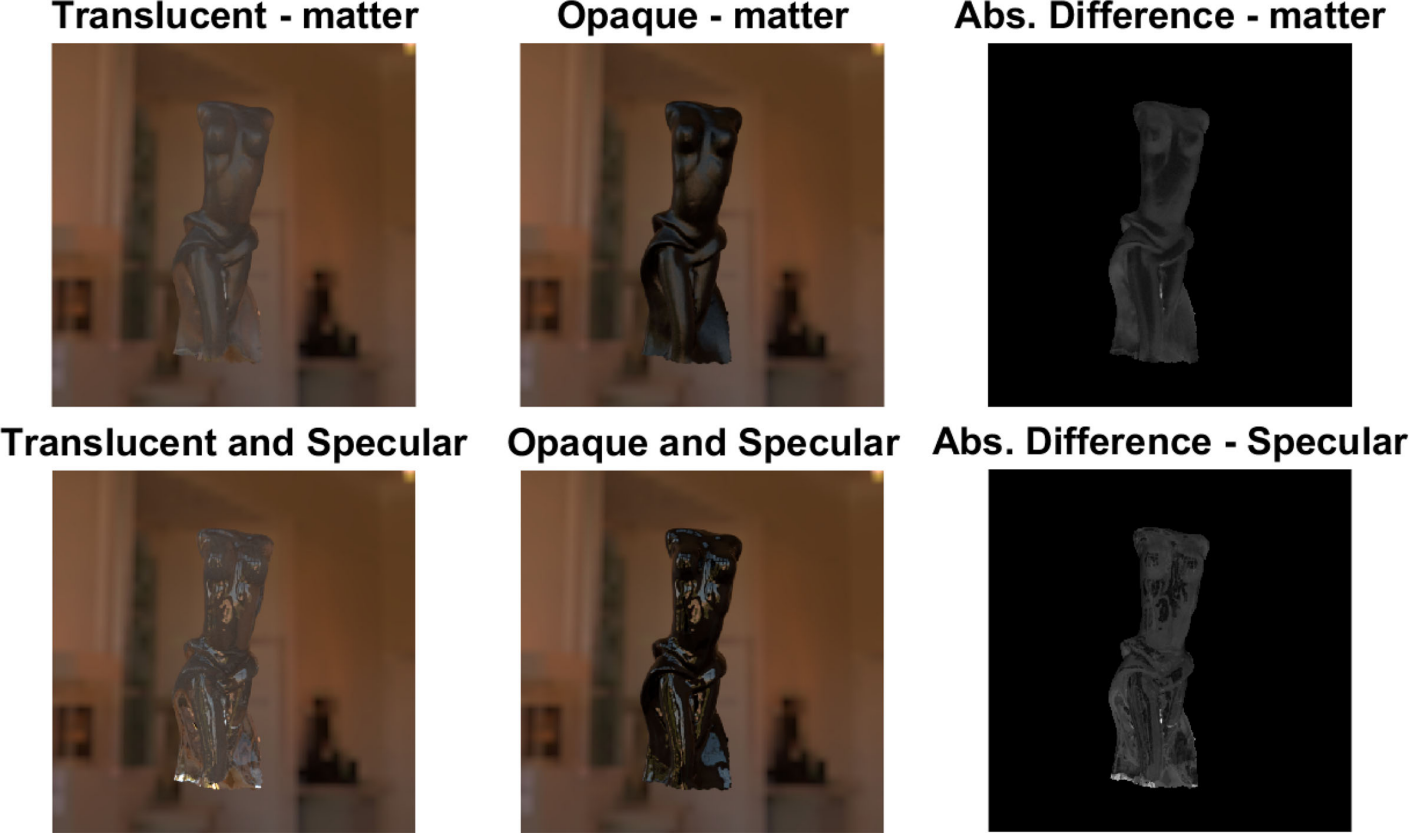
The absolute difference between the two images shows that the translucent and opaque objects have identical intensities in the specular regions. Besides, the contrast between the specular and non-specular regions as well as the spatial coverage of the regions where a translucent object and opaque object differ in intensities is modulated by surface roughness and thus, glossiness of the object.

Motoyoshi (2010) has observed that specular regions remain relatively intact by the subsurface scattering and what varies across different levels of translucency is the appearance of the nonspecular regions. Similarly to the earlier work (Fleming & Bülthoff, 2005), Motoyoshi noted that the non-specular regions usually get blurrier and lighter when subsurface scatter increases. Let's refer to Figure 27, which illustrates the absolute difference between translucent and opaque objects with different levels of specularity. This demonstration supports Motoyoshi's observation that the difference between highly translucent and highly opaque objects is minimal in the areas of specular reflections, which makes us conclude that the spatial regions diagnostic for translucency can be dependent on the surface roughness and the extent of specular coverage.

Furthermore, Motoyoshi also noted similar to Fleming and Bülthoff (2005), that the pixel intensity correlation between translucent and opaque materials is far from linearity, but still not random. They separated the image into different spatial-frequency sub-bands using a Gaussian band-pass filter. Afterward, they manipulated and measured root mean square contrast in each of the frequency domains. The relationship between the contrast in the nonspecular regions and translucency is nonmonotonous. At first, the root mean square contrast decreases as we move from opacity towards translucency. However, for transparent and highly transmissive media, the contrast is either reversed or totally random. This can be attributed to the fact that the contribution of the background increases. This phenomenon can be observed in the thin parts of the dress shown in Figure 11. This is what histogram alone cannot capture without being aware of the spatial information. They have further shown that although the contrast in both low spatial and high spatial domains contribute to translucent appearance, the latter is more important and is able to yield translucent appearance even if the contrast in the low spatial frequency is held constant (refer to Figures 5 and 6 in Motoyoshi, 2010). This observation has implications in the image-based material editing and it has been demonstrated to be important for the image-based translucency transfer (Todo et al., 2019). The observation that blurring nonspecular regions is associated with translucency, while specular highlights remain intact, also explains why N-shaped nonlinearity, which generates larger changes for lower intensity inputs, has been able to enhance the perceived degree of translucency.

### Xiao et al.

**Figure 28.**
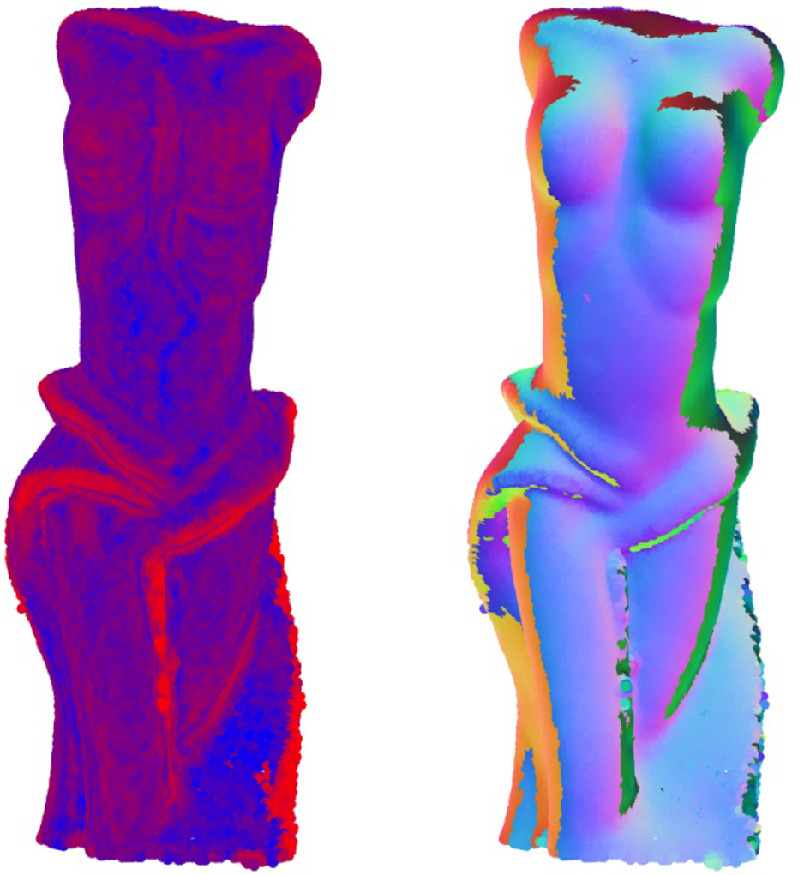
The left figure illustrates the surface curvature of the object (red areas, high curvature; blue areas, low curvature). The curvature can be interesting in two ways: it highlights sharp details and surface concavities which are expected to be in a shadow in case of opaque objects; on the other hand, the sharp edges near the thin areas, which transmit light easily, are also characterized with high curvature. However, note that non-edge parts of the flat thin regions have low curvature, but still transmit large amount of light (see the bottom right corner of the figure; the edge is red, but most of its thin dress part is in blue). The right image is a pseudocolor map of the surface normals; the points where the normals are facing the same direction are colored with similar colors. On opaque surfaces, the normals and thus, the shape can be estimated from the shading information.

Xiao et al. (2020) have shown that a sharp surface relief enhances perceived opacity and argue that this can be attributed to sharper and darker shadows generated by these areas, which on the one hand agrees with the previous findings (Fleming & Bülthoff, 2005; Motoyoshi, 2010) that blurriness and brightness (mean luminance) of the shadowed regions can play a role in translucency perception, but on the other hand, should be taken with care, as the sharp and fine details of the surface can also lead to interreflections and bright appearance, as this is the case for the Lucy in Figure 14. In another work (Xiao et al., 2014), they observed that the thin parts and fine details of the Lucy contribute most to translucent material discrimination, supporting similar observations by other researchers (Fleming & Bülthoff, 2005; Gkioulekas et al., 2015; Nagai et al., 2013). One objective measure for this kind of sharp details could be surface curvature, which to some extent captures both sharp-fine details and outer edges of the object (refer to Figure 28). However, such metric does not capture flat thin areas (see the blue region in the bottom right corner of the figure) that, although have low curvature, still seem to be different, which makes them a diagnostic cue for distinguishing between opaque and transmissive materials.

### Gkioulekas et al.

**Figure 29.**
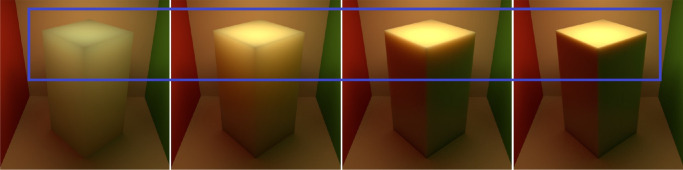
The objects are rendered with a skimmed milk material (Jensen et al., 2001) and an extinction coefficient is scaled to different levels. The intensity distribution at the edges varies considerably between different optical densities. As noted by Gkioulekas et al. (2015) the local maxima are moved closer to the edge, when optical density increases (observe darker strips across the edges in the left two images). However, when optical density is too high (the rightmost image), the material becomes opaque and the facet which is not directly illuminated looks dark and homogeneous. The blue frame highlights the areas where the edges are most informative.

One particular instance of thin regions, the edges, generally have been observed to be informative about material translucence (Fleming & Bülthoff, 2005; Xiao et al., 2014). Therefore, Gkioulekas et al. (2015) tried to take advantage of this and utilized the radiance information near the edges to deduce the subsurface scattering properties of a material. They limited the study to the edges which are the result of surface discontinuity, such as those at the boundary of the two facets of a cube. They split the edges into four qualitative regions on the two facets and simulated a broad range of materials to observe how the radiance information in those regions varies. They found that each material has its surprising “signature” radiance profile at the edges (e.g., refer to Figure 29). Each radiance profile encapsulates information about reflection, refraction, and scattering properties of a material. They analyzed from an optical point of view, how single scattering (single bounce of a photon), mid-order scattering, and high-order scattering contribute to the energy incident on the camera sensor. A typical radiance profile is illustrated in Figure 3 of (Gkioulekas et al., 2015). For instance, a relatively high extinction coefficient puts intensity maxima closer to the boundary on the side facing away from the illumination direction, because the penetration depth decreases (this phenomenon is illustrated in Figure 29 and can be also observed in Figure 3). It is also noteworthy that the high extinction coefficient eliminates the maxima completely due to opacity (Figure 29). High albedo, that is, a higher portion of scattering and a lower portion of absorption in a given extinction coefficient, impacts intensity of these extrema, not their locations. When the albedo is high, high-order scattering contributes more than single scattering and the intensity decreases. The angular variance of the phase function also impacts the location of the local maxima. These local maxima can be noticed as small peaks in Figure 4. Afterward, they demonstrated that different scattering effects can generate matching radiant profiles, that is, edge profile “metamers.” In contrast, when scattering properties are fixed, the profile is unique to a given refraction and illumination direction. We can, indeed, match refraction and illumination effects on the one facet, for example, if the refraction index changes, we can change the illumination angle accordingly to get the “original” reflectance angle; however, this change will impact the other facet, generating a different radiance profile. The idea of using radiance profiles for translucency discrimination was novel. The authors demonstrated that their findings generalize well across many illumination geometries and broad range of translucent materials that makes this work one of the most significant contributions to the topic. In contrast, it is also worth noting that the study was limited to the convex edges and might not generalize to concavities with strong interreflections (such as Lucy in Figure 14).

The authors argued that the edge radiance profiles are robust to the real-world artifacts and can be reliable indicators for edge detection and material identification algorithms in computer vision systems. While they seem a robust indicator for machines, it remains unknown whether the HVS relies on similar edge profiles for translucency perception. Psychophysical experiments need to be conducted in the future to explore this question. It is interesting to observe whether image manipulations and mapping textures of different radiance profiles near the edges of different surfaces affect observers' estimations of the subsurface scattering properties. Additionally, proper eye-tracking measurements could also reveal the saliency of the edge profile components when subjects are performing translucency-related visual tasks.

### Marlow et al.

**Figure 30.**
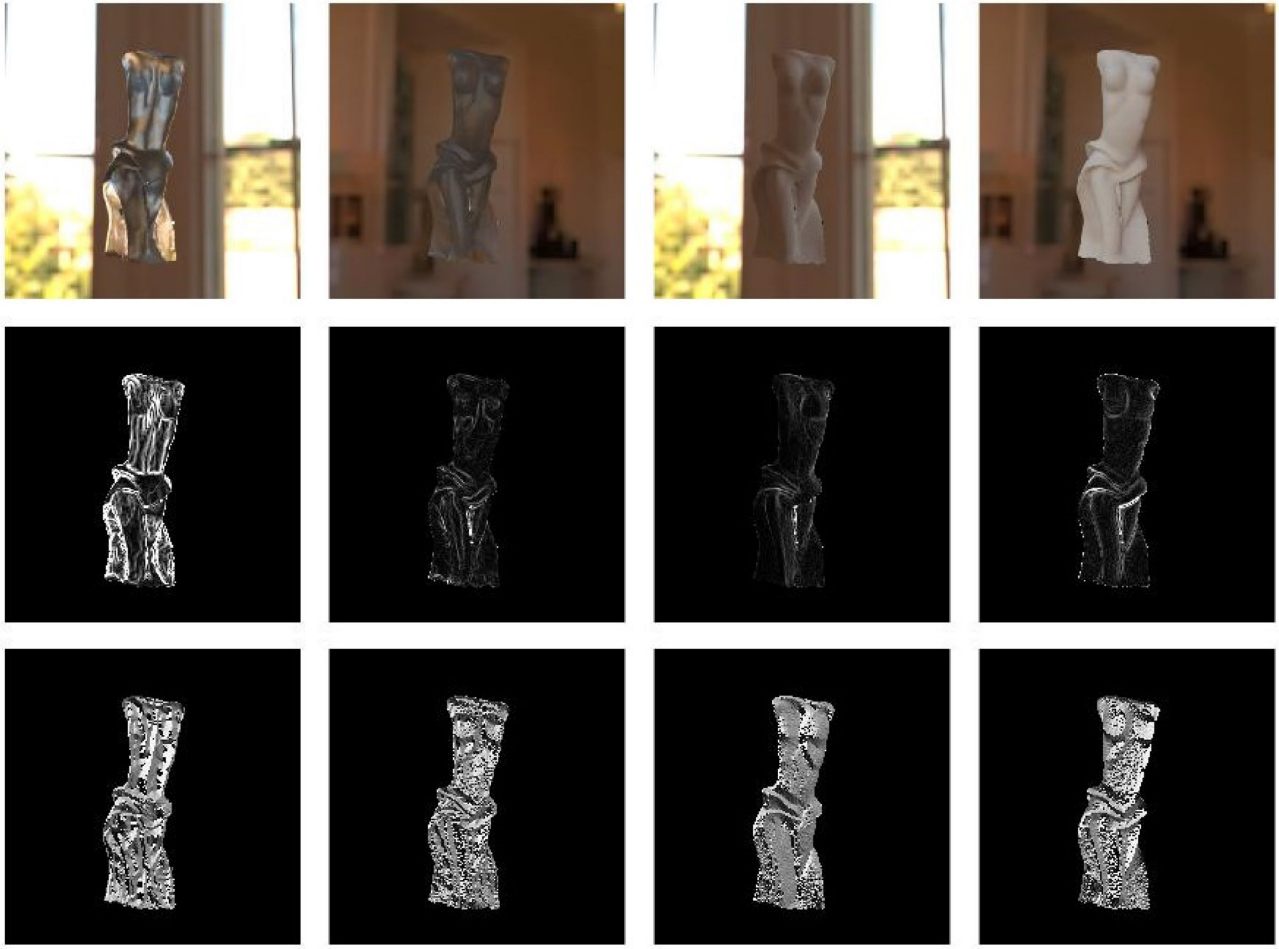
The top row illustrates the original images, the middle row shows the magnitude of the luminance gradient (the background is ignored), and the shades in the bottom row correspond to the gradient orientation. The back-lit translucent object has a high magnitude gradient, whereas it looks relatively homogeneous when front-lit. Under front light, a diffuse object produces more visible gradient (due to shading in the surface convexities) than a front-lit translucent one. In the front-lit condition, the gradient orientation closely follows the surface 3D geometry. When the objects are back-lit, the gradient orientation of an opaque object is strongly impacted by the partial side-illumination, while it looks more random for the back-lit translucent object, which also makes it difficult to recover its shape.

Marlow et al. (2017) have shown that the covariance between surface orientation and shading is related to opacity, while the lack of it produces translucent appearance. They mapped an identical texture of the luminance gradient onto the surfaces with different apparent 3D shapes. They observed that the interpretation of the material properties from a given luminance gradient is impacted by the perceived 3D shape. Particularly, if the image intensities covary with the perceived surface orientation, the material seems to be opaque; otherwise, it seems to be translucent (refer to the Movies S1 and S2 in Marlow et al., 2017). Additionally, they illustrated that when the light field the material is embedded in “accidentally” eliminates this covariance between surface and shading of an opaque object, a vivid and convincing illusion of translucency is observed. The fact that perceived 3D shape impacts the apparent translucency implies that the luminance contrast, mean luminance or similar statistics per se are not enough to explain the perception of translucency, and the HVS is likely to be exploiting surface geometry and 3D shape information as well. However, it is not clear how the HVS calculates the geometry from the retinal images. In our opinion, one way the HVS might be quantifying this is the relation between the surface curvature and the surface normals (Figure 28), on the one hand, and the magnitude and direction of the shading gradient, on the other hand (Figure 30). Although the gradient orientation largely depends on the surface 3D geometry in the diffuse opaque objects, it is more random in objects with a high degree of subsurface light transport. Moreover, the gradient magnitude is largest in highly curved areas in the opaque object, while that is not necessarily true for the translucent ones (see the caption in Figure 30 and compare with Figure 28).

### Marlow and Anderson

Marlow and Anderson (2021) have recently shown that translucent materials are also subject to photogeometric constraints. The authors argue that there is a covariance among the luminance gradient produced by the subsurface scattering of light, the shape of the specular reflections and the shape of the self-occluding contours, and this covariance provides information about material properties and the 3D shape of the object. The covariance can be rooted in the fact that all three components — subsurface scattering, specular reflections, and self-occluding contours, are affected by the same objective geometric priori — the 3D surface curvature. For instance, both the luminance gradient produced by the subsurface scattering and the shape of the specular reflections are usually aligned with the direction of the lowest surface curvature making them aligned with one another as well.

First, the authors demonstrate that the intensity gradient produced by the subsurface scattering is affected by the 3D shape. In opaque materials the location of the luminance extrema depends on the surface orientation in space, as the luminance extrema are located on the sides of the convex and concave regions whichever side faces the illumination is brightest and whichever faces away from it is in the shadow. Contrastingly, in translucent materials, the intensity gradient is related with the local surface curvature; the decrease in the extinction coefficient usually smoothens the gradient, decreases the luminance contrast (which is consistent with other works (Motoyoshi, 2010)) and moves locally brightest and darkest intensities closer to the peaks of the convexities and concavities, respectively. The authors also provide an optical explanation for this: the light attempting to exit the material is redirected towards convexities and away from concavities owing to the internal reflections. These observations on translucent materials generalize well to a broad range of frontal and side illumination angles.

Information about 3D shape can facilitate the estimation of the material properties and vice versa. However, in real-life scenario, neither is hardly ever known to the observer. The HVS somehow manages to recover both 3D shape and properties of a material that according to Marlow and Anderson (2021) can be rooted in the above-mentioned covariation among subsurface scattering, specular reflections and self-occluding contours. The authors conducted psychophysical experiments, which supported those hypotheses. They observed that presence of self-occluding contours and specular reflections increased the vividness of the perceived 3D shape of a bumpy translucent surface. On the other hand, the magnitude of perceived translucency was significantly increased by the specular reflections but was barely affected by self-occluding contours.

This work opens a new avenue for translucency perception research. Although previous work concluded that information on 3D shape is important for translucency (Marlow et al., 2017), this work is the first one to propose that the HVS might be recovering shape and material properties simultaneously, from the same photogeometric constraints. The major limitation of the work is that it does not cover back-lit objects. Identification of the similar photogeometric constraints for back-lit objects is considered very difficult or even impossible by the authors, leaving the question open.

### Di Cicco et al.

Di Cicco et al. (2020c) have recently conducted a study on citrus fruit images. They used multidimensional scaling and constructed a 2D perceptual space explaining the qualities related to translucency. This is an elegant example how translucency perception research can benefit from relying on artworks. They observed that color saturation, intensity gradient and highlights were visible features for translucent materials, while being also related to “juiciness.” They argue that the intensity magnitude and sharpness in their case supports earlier findings that blur and contrast are important cues for translucency perception. The authors also identified *translucency-related regions*, similarly to Nagai et al. (2013), which in their case is the “peeled side” of the fruit. This can be accounted to the fact that the pulp permits light penetration and bleeding around the edges. Although overall trends and cues are consistent with the previous findings, the peculiarity of the stimuli makes generalization to other translucent materials and photorealistic stimuli debatable.

### Summary

A full perceptual model of translucency which could simply take scene and material properties as an input and provide an estimation of a perceptual correlate remains beyond reach nowadays. The possibility of coming up with this kind of model anytime soon ranges from unlikely to impossible. Nevertheless, some partial models have collected interesting observations about translucency perception cues. These works complement each other and can be summarized as follows.

(1) It seems that neither luminance nor spatial information alone is enough for estimating perceived translucency. The HVS seemingly uses some sophisticated combination of the both.
(2) The spatial regions where a photon can go through easily look brighter and contain rich information about material translucence. Examples of this kind of regions are edges, thin parts and sharp fine details of a surface geometry.
(3) The regions which are usually shadowed in opaque objects are also informative about translucency, as they look brighter in translucent materials.
(4) Points 2 and 3 can be generalized as follows: if, in the absence of subsurface light transport, a considerably smaller amount of light could have reached a particular region, this region can be diagnostic for material translucence.
(5) Understanding how much light could or could not have reached a particular region inherently involves understanding the surface geometry and global correlation among different spatial regions.
(6) It is not known how the HVS segments an image, how it identifies the informative regions and how it calculates the surface geometry. These calculations are not unique and vary across individuals. There can be multiple translucency cues in a proximal stimulus and different people can rely on different ones for yet unknown reasons.

We believe that, in addition to standard psychophysics, where experimenters attempt to find a correlation between the varying physical parameters and the observer responses, it is also important to study translucency perception process from a behavioral perspective. The first step toward this has been done by Gigilashvili et al. (2018b, 2021b). In a subsequent section, we analyze what visual mechanisms remain to be uncovered and what factors complicate the translucency perception research.

## Challenges and knowledge gaps

### Inconsistent definition and conceptual ambiguity

The exact meaning of translucency is not universally accepted and remains subjective (Pointer, 2003). This basic definition problem might make the scientific communication difficult and hinder the advance in the translucency perception research. We have particularized these problems in the recent position paper (Gigilashvili et al., 2020b). Care is needed to avoid miscommunication of the empirical results and to ensure the reproducibility of the psychophysical experiments. Experimenters should make sure that the instructions are correctly understood and interpreted by their observers when the task concerns translucency perception, especially when the experiments are conducted in languages other than English, because the translation of the term *translucency* might or might not differ from that of *transparency.* For example, Motoyoshi (2010) reports that there is no distinction between *transparent* and *translucent* in the Japanese language, which might have impacted his experimental results. However, he reports that observers assess translucent and transparent stimuli differently from each other, seemingly understanding the semantic difference between the two visual phenomena. This makes the author propose that the two concepts might be orthogonal. Scaling translucency remains a challenging and confusing task. To the best of our knowledge, Hutchings and Scott (1977) and Hutchings and Gordon (1981) (cited in Hutchings, 2011) have been the first ones to observe the confusion among the experiment participants while scaling translucency. The authors argue that “care should be taken when using the term Translucency for scaling. An increase in translucency may mean an increase in transparency to some panelists while meaning the opposite to others” (Hutchings, 2011). We have also observed a similar kind of problem in our experiments (Gigilashvili et al., 2018b, 2020a, 2021b). The lack of knowledge on how to quantify translucency makes it challenging to measure it by magnitude estimation techniques (Torgerson, 1958) and psychophysical scaling methods, such as the pair comparison and rank order (Engeldrum, 2000). For example, it has been possible to quantify the magnitude of glossiness (Pellacini et al., 2000) or to differentiate more glossy and less glossy stimuli (Gigilashvili et al., 2019b; Thomas et al., 2017). However, there is no universal agreement what “more translucent” means, neither can we tell “how much” translucency is in a given stimulus. When comparing multiple stimuli, which one is the most translucent (e.g., in Figure 2) — the one closest to transparency, closest to opacity or closest to a hypothetical peak between the two? Di Cicco et al. (2020b) have observed that translucency was judged least consistently among all assessed parameters in the still life paintings of grapes, which might be attributed to the variation in the conceptual understanding, rather than the anatomical differences among observers. Nagai et al. (2013) defined *more translucent* in their experiments as having stronger subsurface scattering. Wijntjes et al. (2020) have defined translucency as “the opposite of opaqueness, but... not limiting to pure transparency. For example, tea with milk is more translucent than a cup of white paint.” Di Cicco et al. (2020b) asked observers to quantify the magnitude of translucency of the painted grapes and defined the term in a similar manner: “Translucency: how translucent do the grapes appear to you? Low values indicate that no light passes through the grapes and the appearance is opaque; high values indicate that some light passes through the grapes.” However, care should be taken in these cases as well, because we do not know whether the relation between scattering and translucency is monotonous. Materials with high and low scattering might be considered opaque and transparent, respectively, with both having zero translucency. Many works avoid direct quantification of translucency in the psychophysical experiments and encapsulate it in the matching tasks asking observers to match the stimuli by appearance (Fleming & Bülthoff, 2005; Xiao et al., 2014) and/or by translucency (Gigilashvili et al., 2019c; Gkioulekas et al., 2013; Xiao et al., 2020). This, at first glance, simplifies the task. However, there is little empirical evidence that the HVS can fully isolate translucency from other attributes of total appearance. If the definition of translucency is ambiguous to the observers, how can they match materials by translucency and how can we guarantee that they are not making up their own rules for matching the stimuli, e.g. by lightness, or any property other than translucency? To identify what observers are basing their decisions on, the experimenters can calculate particular image statistics and check how well these statistics explain the variation in the observer responses (as done by Chadwick et al., 2019). However, there is no guarantee that the actual statistics or cues used by observers will be correctly identified by the experimenters. Another workaround found in the literature is using the terms more familiar and less abstract than translucency. For instance, Chadwick et al. (2019) asked observers to assess *strength* and *milkiness* of the tea images. However, the association between the strength, milkiness, and translucency is not clear either. Hutchings (2011) proposes using *extent of visibility* scale of Galvez and Resurreccion (1990) instead of referring to *“more translucent”* and *“less translucent.”* However, the scale is intended for assessing the appearance of the mungbean noodles in a plastic cup and for quantifying the visibility of the objects behind the noodle strands — thus, it is not readily applicable to the solid non-see-through materials. Furthermore, the inconstancy of translucency across different shapes makes it challenging to clearly separate translucency as a property of a given object and as a property of a material the object is made of. We observed (Gigilashvili et al., 2018b, 2019c, 2021b) that human observers find it challenging to compare or match translucency across different shapes for two reasons: first, it is difficult to estimate optical properties of a material and to decouple its visual appearance from the shape-related effects (speaks of the limited ability to “invert optics” as it has been noted previously; Anderson, 2011; Chadwick et al., 2019; Fleming & Bülthoff, 2005); second, the task is inherently ambiguous; translucency cues vary not only between the thick and thin objects, but also between the thick and thin regions of a particular object, making observers uncertain which region to assess and how to come up with a single translucency measure. According to Hutchings (1994), a heterogeneous material might have “more than one colour, perhaps more than one translucency, gloss, or surface irregularity” that no appearance profile system can deal with. The observers in the experiments by Nagai et al. (2013) pointed out that heterogeneous translucency which resulted from a varying shape, complicated the task, but it remained still viable according to the authors. This raises a question: should translucency of a complex-shaped homogeneous material be judged globally for a given object or material, or locally for each specific region of an object?

### Challenges in experimental methods

One of the pivotal limitations of the experimental methods are the constraints related to the visual stimuli selection. Real objects, photographs, or computer-generated imagery can be used to study translucency perception psychophysically. All of these methods come with their advantages and drawbacks, which are summarized in Appendix 1 of Gigilashvili et al. (2021b). We advocate for using physical objects which make the experiments closer to the real-life scenarios, permitting binocular vision, interaction, motion cues, a higher dynamic range, and multisensory information (tactile, auditory, olfactory). We hypothesize that the behavioral patterns applied by observers on physical objects are close to their natural way of making judgments. In contrast, we are aware of the trade-offs. Physical objects are difficult and expensive to model, measure and replicate. The experiments usually take longer (Maloney & Knoblauch, 2020) and the risk of damaging, the unpredictable effects of aging and the limited access across the scientific community hinder the reproducibility of the experiments. A descent alternative that permits interactivity, motion, and binocular cues can be the immersive reality technologies.

A further aspect which is problematic from the experimental point of view is the lack of standardization. Normal conditions for observing translucency and a standard observer are not defined. For instance, the contrast sensitivity and the visual acuity might have a significant impact on the experimental results. However, the viewing conditions, such as the distance and the size of the visual field varies across different experiments which complicates the comparative analyses of their findings.

And last but not least, unlike color vision (Emery & Webster, 2019; Lafer-Sousa et al., 2015), the knowledge about cross-individual differences in translucency perception is virtually nonexistent, because pooled experimental results are usually reported. Chadwick et al. (2018, 2019) have observed that the models explaining the variation in the psychophysical data differ among individuals. Similar cross-individual differences were also observed by Nagai et al. (2013) and Gigilashvili et al. (2018b). Whether this could be attributed to the interpretation of the task, prior experience or anatomical differences, need to be answered in the future. Should we expect the translucency counterpart of *#TheDress* anytime soon, which could expose these individual differences?

### Visual mechanisms of translucency perception

The exact mechanisms of translucency perception remain largely unidentified. After compilation of the state-of-the-art works, we came up with the several important questions which we believe should be addressed in future works.

• Which image cues and regions does the HVS rely on and how does it identify, calculate and weight them?
• What is the role of shape and geometry perception and how does the HVS calculate them?
• To what extent is perceived translucency impacted by other appearance attributes, such as color, gloss, texture and fluorescence?
• What role do the identification of the familiar materials and other psychological priors play in translucency perception?
• How does the HVS use motion and scene dynamics to assess translucency?
• What is the physiology of translucency perception from the retinal to the cortical level and how much does it vary across individuals?

We envision that the future work can develop in three directions: the eye-tracking experiments can facilitate identification of the respective cues and key image regions; behavioral analysis (similar to Gigilashvili et al., 2021b) might reveal how the judgments on translucency are made and which factors guide observers’ actions; while neuroscience can shed light to the physiological and cognitive aspects in the perplexing process of translucency perception.

Eye-tracking can potentially reveal the most salient cues to translucency and whether different observers rely on different cues, as noted by Nagai et al. (2013). Eye tracking is a more straightforward way than reverse correlation techniques used by Nagai et al. (2013) to learn where observers look in the process of translucency assessment. Additionally, the saccade paths measured with the eye-tracking could reveal the sequence and the frequency of inspecting particular local regions. This could potentially reveal how different local regions relate to one another. In contrast, we understand that eye tracking comes with the considerable limitations: the reason of the fixations might be unrelated to translucency — some regions might be salient for other reasons, for example, a human face can attract extra attention regardless the task; we will not capture the influence of the parafoveal vision and the cues which are not locally defined; the temporal resolution of the eye tracking equipment might be lower than the speed of the visual processing; the presence of eye tracking equipment might affect the naturalness of the interaction.

## Summary and conclusions

We have discussed translucency as one of the pivotal appearance attributes, which is increasingly important in a broad range industries and disciplines, including 3D printing, cosmetics, the food industry, and the arts, among many. Translucency results from the subsurface transport of light. Although the techniques for measuring and modeling the optical properties of a material are relatively well-established, our understanding how they link to their perceptual correlates remains limited. The advance in translucency perception research is attributed to the development of computer graphics techniques which permit easier generation of the translucent visual stimuli. Although the initial studies were limited to transparency perception, transparency models could not explain the perception of highly scattering media. The visual cues and perceptual mechanisms seem to be fundamentally different between the transparency and translucency of the see-through filters and translucency of highly scattering, non-see-through media. This resulted in emergence of a separate research topic, namely, the perception of translucency in highly scattering media. In the past 20 years, multiple factors have been identified to be contributing to perceived translucency, such as the illumination direction, structural thickness of the object, as well as subsurface scattering properties. It is believed that the luminance distribution around the edges and in the shadowed regions, and its covariance with the surface geometry, might be used by the HVS to infer translucency in highly scattering, non-transparent materials, while the HVS relies on apparent contrast and blur when the background is visible. Nevertheless, overall translucency perception research is still in its infancy. We argue that the problems with the conceptual understanding and comprehension of the term impede the advance of the research and complicate the reproducibility of the tasks. We argue for the better standardization in this domain. Finally, we believe that eye tracking experiments could reveal which image regions and cues are significant, and advance in neuroscience could provide a deeper insight in the corresponding anatomical mechanisms for translucency perception.

## Supplementary Material

Supplement 1

Supplement 2
